# Cross-sectional study on the association between seroprevalence among children and their parents’ knowledge, attitudes, and practices in a dengue-endemic city in Bangladesh

**DOI:** 10.1371/journal.pntd.0014802

**Published:** 2026-10-21

**Authors:** Claudia Ximena Robayo Gonzalez, Bouchra Nasri, Marie-Catherine Gagnon-Dufresne, Avijit Saha, Laura Pierce, Mushfiqur Rahman, John Barnard Mackfield, Yang Zhang, Mrittika Barua, Md. Mizanur Rahman, Malay Kanti Mridha, Valéry Ridde, Kate Zinszer

**Affiliations:** 1 École de Santé Publique, Université de Montréal, Montréal, Canada; 2 Centre de Recherche en Santé Publique, Montréal, Canada; 3 BRAC James P Grant School of Public Health, BRAC University, Dhaka, Bangladesh; 4 Department of SBS, School of Public Health, Virginia Commonwealth University, Richmond, Virginia, United States of America; 5 Department of Health Policy, Vanderbilt University Medical Center, Nashville, Tennessee, United States of America; 6 Center for Non-communicable Diseases and Nutrition, BRAC James P Grant School of Public Health, BRAC University, Dhaka, Bangladesh; 7 Université Paris Cité and Université Sorbonne Paris Nord, IRD, Inserm, Ceped, Paris, France; WRAIR, UNITED STATES OF AMERICA

## Abstract

**Introduction:**

Bangladesh is experiencing a drastic increase in dengue infections since 2019, with a record number of cases exceeding 300,000 in 2023 and 102,082 cases in 2025. Control of dengue, spread by *Aedes* mosquitoes, requires an in-depth understanding of knowledge, attitudes, and practices (KAP) regarding prevention strategies and their association with the risk of infection. This association has been rarely examined in adults and even more rarely in relation to the risk of infection in children. This study aimed to investigate the association between parents’ knowledge, attitudes, and practices (KAP) and children’s dengue seropositivity in Dhaka, Bangladesh.

**Methodology:**

A cross-sectional study was conducted in four neighborhoods of Dhaka in November and December 2023. A total of 297 parents and 412 children aged 2–12 years participated in the study. Parents completed a questionnaire that collected data on their previous dengue experiences, sociodemographic characteristics, social capital, and knowledge, attitudes, and practices (KAP) related to dengue. Children’s capillary blood samples were collected via finger prick and tested for IgM and IgG dengue antibodies. The relative risk (RR) of dengue seropositivity was estimated using a modified Poisson regression model with robust error variance.

**Results:**

The median ages of the parents and children were 32.5 and 6.7 years, respectively. The adjusted seroprevalence of dengue for both IgM and IgG in children was 14.0% (95% CI 10% -19%). Parents’ KAP scores were classified as intermediate knowledge of the disease in 60.3% of participants; 64.3% reported positive attitudes towards dengue control and prevention actions; and 62.0% had a high score for dengue prevention practices. A higher parent attitude score was associated with lower child seropositivity (aRR 0.77, 95% CI 0.60-0.99).

**Conclusions:**

These findings provide preliminary insight into the factors associated with dengue seropositivity in children in Dhaka, offering additional information about parents’ attitudes towards dengue risk and prevention and the relationship with child’s seropositivity. This information can guide public health interventions, such as dengue control campaigns. A more in-depth exploration of the context will help to understand the cultural and social factors that shape KAP in Dhaka.

## Author summary

Dengue is an infectious disease transmitted by mosquitoes and found in tropical and subtropical regions worldwide, such as Bangladesh. It presents symptoms including fever, pain behind the eyes, and muscle and joint pain, which can progress to abdominal pain and bleeding, and may become life-threatening. Children are at higher risk of contracting the infection and requiring hospitalization. Our study included 297 parents of 412 children aged 2–12 years in Dhaka, Bangladesh. Our objective was to understand how knowledge, attitudes, and practices regarding dengue may influence the risk of infection in children. We conducted a survey of parents’ knowledge, opinions, and practices regarding dengue, its control, and prevention, and we administered a dengue screening test to children to determine if they had previously been infected with dengue. Overall, we found that positive attitudes toward prevention practices and the perception of dengue as a serious illness were associated with reduced risk of infection in children. Additionally, participants demonstrated knowledge about dengue and recognized prevention practices. These results suggest that other factors, such as attitudes or perceptions, may influence the risk of infection in this population.

## Introduction

Dengue virus occurs in tropical and subtropical areas, infecting 390 million people from 129 countries annually [1–3]. The global occurrence of dengue has increased steadily since 2000, when there were just over 500,000 cases, compared to 14.6 million in 2024 [1]. Along with the rapid increase in cases, the geographic distribution of dengue is also shifting due to population growth, globalization, climate change, urbanization, and ineffective vector control strategies [2,4].

Bangladesh, located in South Asia, has experienced a substantial increase in the burden of dengue in recent years. In 2023, a record number of cases was reported, with over 300,000 cases, 18% of which were in children aged less than 15 years [5,6]. The largest city, Dhaka, a megacity with a population of approximately 23 million, accounts for 52.8% of cases and 78.9% of deaths in Bangladesh in 2023 [6–8]. Today, dengue remains a problem in the country, with 102,082 cases reported as of December 2025 [9]. The causes of recent dengue outbreaks in Bangladesh are likely due to multiple factors, including the prolonged rainy season, unplanned rapid urbanization, high population density, inadequate clinical and public health infrastructure, suboptimal vector-control programs, and insecticide resistance [10–14]. An additional factor for the 2023 outbreak was a shift in dengue serotype with the re-circulation of DENV-2, associated with more symptomatic and severe cases [15]. The Directorate General of Health Services (DGHS) has implemented dengue control and prevention measures, including reorganizing health services and large-scale education campaigns [5,16], while the Local Government Engineering Department has led vector control activities such as insecticide spraying to control infection [16].

Dengue has four distinct serotypes (DENV 1–4), all of which have historically circulated in Bangladesh [2,14,17]. Each serotype confers serotype-specific immunity [2] and some are associated with severe infection [15,18,19]; however, secondary infections with a different serotype increase the risk of severe disease due to antibody-dependent enhancement (ADE), which enhances viral replication instead of controlling it [2,20,21]. The development of the immune response is a key indicator of infection [22]. Immunoglobulin M (IgM) antibody peaks approximately 14 days after the onset of symptoms and generally remains detectable for two months [2,23]. Immunoglobulin G (IgG) levels increase at the end of the first week of symptoms and remain detectable throughout the patient’s lifespan [21,23]. During subsequent dengue infections, antibody levels increase more quickly, with IgG becoming detectable in the acute phase [21,23]. Dengue infections can be classified as asymptomatic, symptomatic, and severe [24]. Studies show that dengue severity is linked to age [14,22], with children being more likely to develop severe dengue with a fatality rate of 26% among severe cases [22]. A significant proportion (40%) of dengue infections in children are asymptomatic [25,26], which is an important hidden reservoir for dengue transmission. The country’s immunological and seroprevalence data indicate that 54.9% of children under 12 years and 83.1% of adults were IgG-positive in 2017, whereas in 2023 the reported seroprevalence among hospitalized children was 42.6% [27,28]. However, immunological data on symptomatic and asymptomatic cases in children are scarce in the country.

Regarding dengue control, one measure is to focus on community-based interventions that promote community members’ active participation in different activities [29]. The effectiveness of these activities depends on human behavior, for example, the need for community participation in the prevention or elimination of mosquito breeding grounds. The introduction of new activities, such as the coverage and cleaning of water storage containers, requires acknowledging the enablers, barriers, and social norms that facilitate behavioral change for both the individual and the community [30]. An approach to examining the possible implementation of these activities is by using knowledge, attitudes, and practices (KAP) questionnaires [31]. KAP questionnaires are widely used as tools to compile structured information about what people know, believe, think, and do regarding dengue (or other health conditions) at the individual and community levels, and can be linked to the theory of planned behavior (TPB) [31,32]. This strategic alignment may enhance the relevance and acceptability of the interventions, fostering more meaningful and sustainable community engagement [33,34], ultimately reducing dengue transmission.

Studies in Bangladesh evaluating KAP in relation to dengue generally show positive attitudes towards mosquito control practices [35], positive associations between dengue knowledge and the level of education [36], and a lack of association between knowledge and practices [37]. One study in Bangladesh evaluated KAP among hospitalized patients with dengue, finding that those hospitalized for more than 7 days had positive attitudes toward dengue prevention practices [38,39]. Yet, no studies in Bangladesh have examined parental KAP or its potential association with children’s seropositivity. We hypothesize that seropositivity among children in a hotspot area may be related to varying levels of parents’ knowledge, attitudes, and practices regarding dengue. Thus, this study aimed to investigate the association between parents’ KAP and children’s seropositivity in four neighborhoods in Dhaka, Bangladesh.

## Materials and methods

### Ethics approval and consent to participate

This study was approved by the Comité d’éthique de la recherche en sciences et en santé (protocol registration CERSES 2023–4911) at Université de Montréal in Canada, and by the Institutional Review Board at BRAC James P Grant School of Public Health (BRAC JPGSPH), BRAC University in Bangladesh (protocol registration IRB-13 June’23–022). The study and implications of participating were explained to the participants, and a trained research assistant provided an information sheet. Written informed consent (signature or fingerprint) was obtained in the Bengali language.

### Study area

Dhaka, the capital of Bangladesh, is a megacity with a population of approximately 23 million within its metropolitan area [40]. The city is among the most densely populated in the world, with 23,234 people/km^2^ [41]. It is divided into two city corporations, Dhaka North City Corporation (DNCC) and Dhaka South City Corporation (DSCC), each with 54 and 75 wards, respectively [39,42]. A ward is the smallest administrative unit within a local government area, used to elect local representatives and to organize local governance activities [42]. Within these two administrative areas, four neighborhoods were selected. Uttara and Mirpur are located in DNCC, and Chawkbaazar and Mugda are located in DSCC (Fig 1). The neighborhoods were selected based on accessibility and the high incidence of dengue cases reported in 2023 [43,44]. Their selection was based on the need to understand the various geographical challenges, the local socioeconomic context, and the structural diversity of buildings and streets in informal settlements and low- and middle-income areas in Dhaka, providing feasibility information for a larger study [45]. Participants in Uttara and Mugda were predominantly working-class individuals from low-income areas, including house workers. In Mirpur, all participants lived in an informal settlement, also known as a slum, whereas in Chawkbazaar, they were from low- and middle-income areas. In these four neighborhoods, four types of houses are found [46,47]. The first is independent houses that stand alone and are built on separate plots of land, constructed from durable materials. The second is multi-storied buildings, which are residential buildings with multiple floors, accommodating several families in individual units or apartments, constructed of concrete or brick. Then there are the semi-pucca houses, which combine natural materials (mud, bamboo, etc.) with more permanent materials (brick), typically one-story. Lastly, informal settlement houses, which sometimes are made of less durable materials, such as tin [47,48].

**Fig 1:**
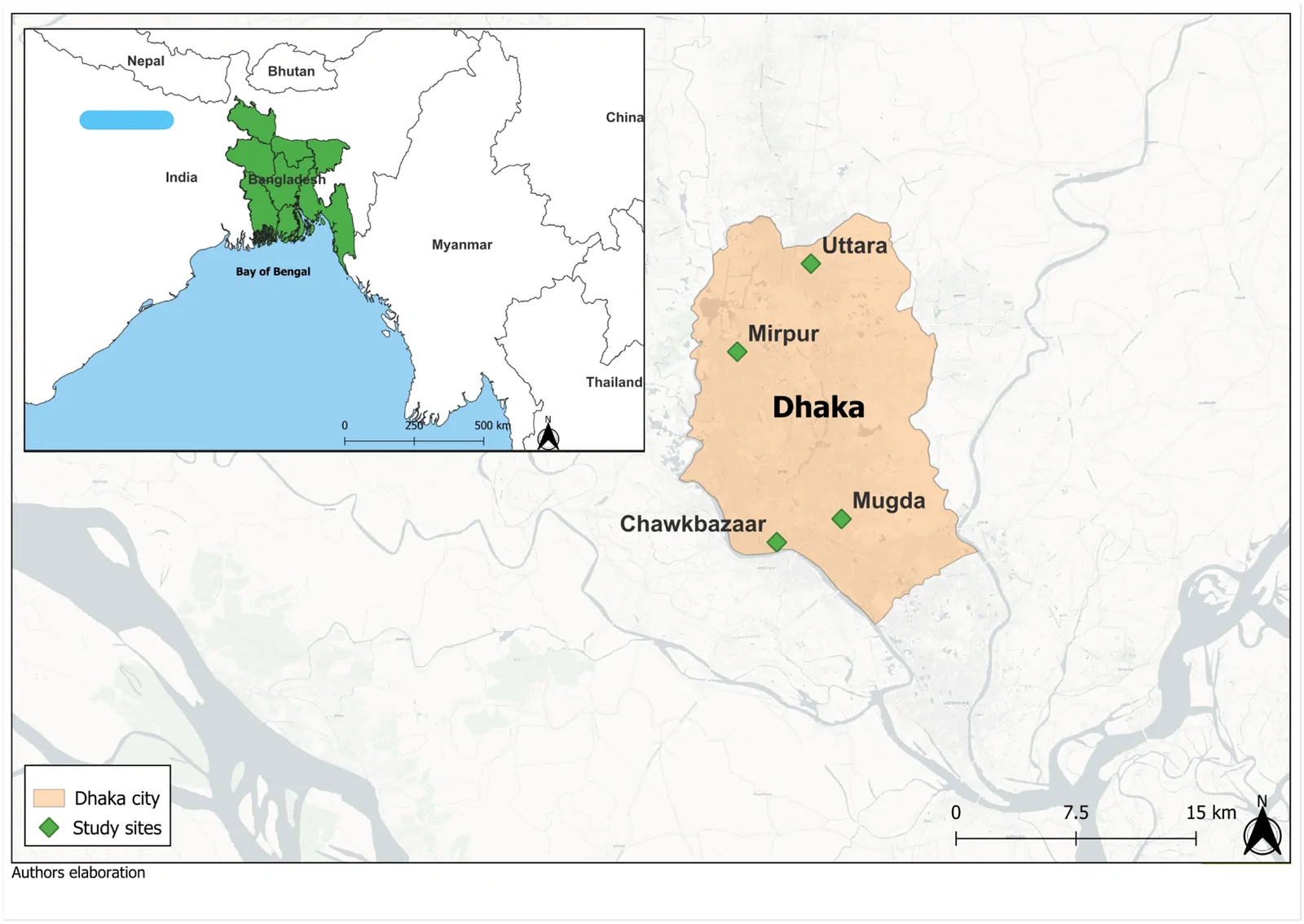
Geographical locations of the study sites. Map created using QGIS (version 3.44) (QGIS.org, 2026. QGIS Geographic Information System. QGIS Association. http://www.qgis.org). Sources: Main panel: Dhaka Metropolitan boundary from OpenStreetMap contributors via Overpass Turbo (https://overpass-turbo.eu/), ODbL (https://www.openstreetmap.org/copyright); basemap titles by CartoDB, CC BY 3.4. Inset: world boundaries from Natural Earth (https://www.naturalearthdata.com/), public domain; Bangladesh boundaries from Simplemaps.com (https://simplemaps.com/gis/country/bd), CC BY 4.0.

### Study design

A cross-sectional pilot study was conducted to inform an upcoming large-scale cluster- randomized controlled trial (cRCT) in Dhaka, Bangladesh. This trial aims to evaluate a community mobilization approach to dengue control [45]. Data collection for this pilot study occurred between November and December 2023, with the objective of assessing the association between parental KAP and dengue seropositivity in children. Secondary objectives included examining the different domains of KAP and their associations with one another. As no standard sample size formula is available for relative risk estimation via modified Poisson regression in cross-sectional cluster sampling, a sample size assessment was performed using standard logistic regression [49]. The sample size calculation for the pilot study was done based on a logistic regression with an odds ratio (OR) reaching 1.8 from previous studies [50,51] and a 15% probability of the outcome in children [6], a statistical power of 0.80, and a significance level of 0.05, which resulted in a sample size of 229 participants.

### Participant eligibility and data collection procedures

Data were collected using a household questionnaire and a rapid diagnostic test for dengue. Household selection followed a systematic approach consistent with methods described for community health surveys [52,53]. Trained enumerators began at a fixed reference point at the geographical center of each neighborhood and proceeded radially through contiguous blocks, selecting every third household, with eligibility assessed at each visited household [52,53]. Apartment blocks were included. Households were eligible to participate if they had children aged 2–12 years living in the household, the family intended to continue living in the area for the next 6 months (as the initial plan was to conduct a second data collection which was later dropped due to political instability in the region), and the parent or legal guardian (hereafter referred to as “parent”) provided consent. For the finger prick, parents gave consent, and the child gave assent before the test. Children with chronic diseases associated with excessive bleeding or with fever at the time of sampling were excluded. Informed consent was obtained by having the person responsible for the household sign or fingerprint. A maximum of two children per household was selected to keep the balance and decrease clustering.

The questionnaire was reviewed by national researchers and adapted to the local language and study context. The questionnaire was administered to parents in Bengali by trained research assistants during door-to-door visits, using the Kobo-Collect tool. The instrument included questions on sociodemographic characteristics, household history of dengue, social capital, and a KAP questionnaire on dengue (S1 Material).

### Knowledge, attitudes, and practices

The KAP portion of the questionnaire consisted of 15 questions, eight on knowledge, three on attitudes, and four on practices. The KAP questionnaire was previously used in a similar dengue study in Brazil [54]. The questionnaire was reviewed and content-validated by local researchers, who adapted it in terms of language and content. Translation and retranslation from English to Bengali were then performed. The questionnaire was validated during the study with a Cronbach’s alpha of 0.54. The three dimensions reflect the KAP model, grounded in the TPB [31]. The questionnaire’s attitudes and practices dimension operationalizes the TPB proximal constructs (attitude toward the behavior, subjective norm, and perceived behavioral control) and the final criterion of behavior itself [55,56]. Knowledge was measured as a dimension connected to both attitudes and practices, rather than a TPB core construct. The knowledge questions addressed modes of dengue transmission, infection, types of symptoms, mosquito breeding sites, information sources, and knowledge of various prevention measures. Regarding attitudes, questions were asked about perceptions of infection risk and opinions related to preventive measures. In the practice section, questions focused on actions for dengue prevention and mosquito control from the individual and the city corporation. The questions in the knowledge and practice domains were multiple-choice and yes/no questions. For the attitude domain, all questions were on a five-point Likert scale, ranging from “completely agree” to “completely disagree” (S1 Material).

A score was created for each question. Multiple-choice questions with a single answer were assigned one point for each correct answer and zero points for incorrect or “don’t know” answers. Multiple-choice questions with multiple answers were assigned one point for a first correct answer and 0.5 additional points for each correct secondary answer. For the Likert-type questions, a score of 2 was assigned to responses with the highest level (completely agree), decreasing by 0.5 with each choice to a score of zero for the “completely disagree” or “do not know”. Then, the domain scores were summed, with a maximum of 14 for knowledge, 6 for attitudes, and 7 for practices. Each of the domains and the total score was categorized into three levels (less than 50% of points, between 51 and 75% of points, and greater than or equal to 75% of points), following the Bloom recommendations [57]. This procedure was followed because there are no specific guidelines for creating levels for KAP scores, and Bloom’s cutoffs have frequently been used in KAP studies, providing a theoretically grounded procedure comparable to other studies [58,59].

### Seroprevalence test

Children’s blood samples were collected via a finger prick by a trained technician during the questionnaire administration and immediately analyzed using the SD Bioline Dengue Duo Rapid Diagnostic Test (RDT). The RDT was selected for its practicality in field-based data collection, providing rapid, easy-to-interpret screening for dengue [60,61]. and being appropriate for a pilot study without laboratory infrastructure at the point of collection. This test detects antibodies for IgM, IgG, and NS1 antigens for all four dengue serotypes. Reported sensitivities compared to ELISA vary across settings, ranging from 50.75% to 69.78% for IgM and from 69.03% to 85.35% for IgG [60,61], while the manufacturer reports sensitivities of 94.2% for IgM and 96.4% for IgG [62]. The test required 15–20 minutes to produce results, which were presented immediately to the adult participant with appropriate interpretation provided by the field staff. Seropositivity was defined as a positive test for IgM, IgG, or both [60,62].

### Outcome: Seropositivity

The outcome was seropositivity, defined as positive for IgM, IgG, or both. IgM detection alone was not used, given the limited sensitivity of rapid diagnostic tests for IgM without NS1 antigen confirmation, which was unavailable in our study [61]. Given that IgG can remain detectable for an extended period following infection, this combined outcome reflects evidence of current or past dengue infection among children [21,23]. Seroprevalence was estimated using a single-proportion z-test with 95% confidence intervals. Adjusted estimates for the seropositivity were done using the sensitivity and specificity of the test (S2 Material) [61].

### Exposure: KAP of the parents

The exposure variable was the KAP score (as previously described). Knowledge was a continuous variable with a maximum score of 14 points, categorized into levels: essential (<7 points), intermediate (7- < 10.5 points), and knowledgeable (≥10.5 points). For attitudes, there was a maximum of 6 points, with levels of negative (< 3 points), neutral (3- < 4.5 points), and positive attitudes (≥4.5 points). For practices, the maximum score was 7, categorized as introductory practices (<3), partial (2- < 5.25), and extensive (≥5.25). As part of the evaluation, a total KAP score was calculated as the sum of the domain scores (0–27). This was categorized as low (<13.5), moderate (13.5- < 20.25), or high (≥20.25).

### Covariates

Included covariates and confounding variables were selected based on prior evidence and after constructing a Direct Acyclic Graph (DAG) [27,39,54,63–66]. The selection of these variables was based on studies that reported positive or negative associations with seropositivity. The covariates considered were as follows: age of the parent (continuous), gender of the parent and the child (categorical), more than 5 years living in the current house (categorical), occupation (categorized into working and not working), type of water storage (closed/no stored, or open), household income index (categorized into low, moderate and high to reflect the income of the household), and social capital index (dichotomized into low/moderate vs. high social capital). Likewise, the age of the child (continuous), parent level of education (categorical, less than primary school), previous dengue episodes of the child in 2023 (categorical), previous hospitalizations due to dengue in the house (categorical), and number of people in the house with the dengue episode in 2023 (categorical) were incorporated as possible confounding factors identified in previous studies.

To construct the household income index, 14 variables recommended by previous studies were used, reflecting construction materials, house type, sewage system type, number of rooms, appliances, and outdoor areas [67,68]. These variables were used because they represent not only the house material but also other assets, such as televisions, refrigerators, and fans [69]. Each question was individually scored based on its answer options (S1 Material). The points were added to the total score and divided into three quantiles: low, moderate, and high. Similarly, the social capital index was constructed based on the approaches of Alvarado-Castro [70] and Siegel [71]. Factor analysis was performed to identify which questions to include in the index and how many factors to extract. Four factors were identified, grouping 10 of the questions. Questions 7, 9 and 13 were not included as they did not group into any factor according to the approach (S1 Material). A total score of 14 was divided into low/moderate (<7 points) and high (≥7 points) [70].

### Statistical analysis

Descriptive analyses of the KAP questions were performed, stratified by neighborhood, using measures of central tendency and absolute and relative frequencies. To test the correlation between KAP domains and seropositivity, we performed a chi-squared test. The correlation between the domains of KAP was assessed using Kendall’s rank correlation (τ), selected due to the ordinal nature and non-normal distribution of the data. The strength of the correlation was interpreted according to the absolute values of τ: absent (=0), weak (0.1–0.3), moderate (≥0.3–0.5), and strong (≥0.5) [72,73]. The association between KAP and seroprevalence was initially evaluated using univariate regression models, as there is no standard recommendation for including KAP in regression analyses (continuous variable, categorical levels, total score, or by domain). These combinations were tested in the univariate regression models between parents’ KAP and children’s seropositivity (S4 Material).

Subsequently, a multivariable regression model was constructed to analyze the relationship between seropositivity and KAP score, with other covariates of epidemiological importance according to the DAG. A modified Poisson regression model was used instead of logistic regression, given the prevalence of the outcome greater than 10% [74,75]. Taking into account the potential correlation in the data due to belonging to the same neighborhood and/or household, a robust error-variance estimation was performed instead of multilevel analysis [75]. This decision was based on the small number of clusters (four neighborhoods) and the restricted number of participants per household (maximum two per household). The number of clusters and the number of participants per group in this study fell below the minimum average required for reliable multilevel parameter estimation (10–15 clusters and an average of five participants per group) [76,77]. Under these conditions, multilevel analysis produces biased variance estimates and poor confidence interval coverage, whereas a robust variance estimator empirically accounts for within-cluster correlation [77]. This robust error variance also addresses the overly conservative standard errors of the Poisson model when applied to binary data, minimizing the loss of statistical power relative to the original sample size calculation for logistic regression [74,75]. Neighborhood was therefore included as a fixed effect rather than modeled as a random effect, and dependence between individuals within the same household was addressed using a robust variance estimator rather than a household-level random effect. Collinearity was assessed using the variance inflation factor (VIF), considering values >2 as indicative of collinearity. There was no missing data in the questionnaires. Statistical analyses were performed using R version 4.4.3.

## Results

### Characteristics of parents, children, and households

The study approached 1,226 households, of which 447 did not have a child, 3 did not have a child between 2 and 12 years, 167 had a child but opted not to participate, and 103 had no informed respondent. Of the 506 households with the inclusion criteria, only 328 were willing to participate. Of those, 30 did not have a household head at the time of data collection. One household could not participate because the child was older than 12 years at the time of data collection. The study included 297 parents and 412 children. Participants from the Mirpur neighborhood lived in an informal settlement, in semi-pucca houses. Chawkbazaar participants lived in multi-storied houses (Table 1). Accordingly, the highest income index was found in 71.2% of the homes in Chawkbazaar, followed by Mugda (49.2%). The social capital index, which accounts for community participation, was not significantly different among the four locations.

**Table 1:** Characteristics of adults, children, and households.

Responding parent’s characteristics	Uttara	Mirpur	Mugda	Chawkbazaar	Total
	(N = 85)	(N = 81)	(N = 65)	(N = 66)	(N = 297)
**Gender**					
Male	12 (14.1%)	12 (14.8%)	12 (18.5%)	13 (19.7%)	49 (16.5%)
Female	73 (85.9%)	69 (85.2%)	53 (81.5%)	53 (80.3%)	248 (83.5%)
**Age (years)**					
Median [Min, Max]	30.5 [20.0, 51.1]	35.2 [18.1, 77.2]	32.0 [18.9, 51.1]	31.5 [20.1, 81.3]	32.5 [18.1, 81.3]
**Literacy**					
Can read and write	57 (67.1%)	48 (59.3%)	49 (75.4%)	53 (80.3%)	207 (69.7%)
Can only read	0 (0%)	0 (0%)	2 (3.1%)	1 (1.5%)	3 (1.0%)
Cannot read and write	28 (32.9%)	33 (40.7%)	14 (21.5%)	12 (18.2%)	87 (29.3%)
**Level of education**					
Primary school or less	53 (62.4%)	55 (67.9%)	29 (44.6%)	24 (36.4%)	161 (54.2%)
Secondary school finished or unfinished	28 (32.9%)	24 (29.6%)	32 (49.2%)	37 (56.1%)	121 (40.7%)
Diploma/Vocational	1 (1.2%)	0 (0%)	0 (0%)	2 (3.0%)	3 (1.0%)
University	2 (2.4%)	0 (0%)	4 (6.2%)	3 (4.5%)	9 (3.0%)
Hafezi/ Qawmi/ Kharizi *	1 (1.2%)	2 (2.5%)	0 (0%)	0 (0%)	3 (1.0%)
**Working status**					
Working currently	47 (55.3%)	33 (40.7%)	25 (38.5%)	21 (31.8%)	126 (42.4%)
Household work/housework	27 (31.8%)	33 (40.7%)	39 (60.0%)	44 (66.7%)	143 (48.1%)
Does not work	11 (12.9%)	15 (18.5%)	1 (1.5%)	1 (1.5%)	28 (9.5%)
**Number of people in the household with self-reported dengue episodes since May 2023 (not including participating children)**					
Nobody in the house was diagnosed with dengue	62 (72.9%)	72 (88.9%)	51 (78.5%)	63 (95.5%)	248 (83.5%)
One or more	23 (27.1%)	9 (11.1%)	14 (21.5%)	3 (4.5%)	49 (16.5%)
**Self-reported dengue episodes in the lifetime of family members**					
No one has had dengue	70 (82.4%)	71 (87.7%)	41 (63.1%)	49 (74.2%)	231 (77.8%)
Yes, at least one person	15 (17.6%)	10 (12.3%)	24 (36.9%)	17 (25.8%)	66 (22.2%)

**Child characteristics**	**Uttara**	**Mirpur**	**Mugda**	**Chawkbazaar**	**Total**
	**(N = 115)**	**(N = 114)**	**(N = 90)**	**(N = 93)**	**(N = 412)**
**Gender**					
Male	57 (49.6%)	59 (51.8%)	49 (54.4%)	41 (44.1%)	206 (50.0%)
Female	58 (50.4%)	55 (48.2%)	41 (45.6%)	52 (55.9%)	206 (50.0%)
**Age of the child**					
Median [Min, Max]	6.95 [2.00, 12.9]	6.92 [2.05, 12.9]	6.12 [2.09, 12.9]	6.24 [2.01, 12.6]	6.74 [2.00, 12.9]
**School attendance**					
Yes	61 (53.0%)	69 (60.5%)	54 (60.0%)	46 (49.5%)	230 (55.8%)
No	54 (47.0%)	45 (39.5%)	36 (40.0%)	47 (50.5%)	182 (44.2%)
**Chronic disease**					
Yes	0 (0%)	0 (0%)	4 (4.4%)	5 (5.4%)	9 (2.2%)
No	115 (100%)	114 (100%)	86 (95.6%)	88 (94.6%)	403 (97.8%)
**Parent-reported previous dengue episode since May 2023**					
Yes	4 (3.5%)	0 (0%)	9 (10.0%)	4 (4.3%)	17 (4.1%)
No	111 (96.5%)	114 (100%)	81 (90.0%)	89 (95.7%)	395 (95.9%)
**Seropositive (n = 333)**					
Yes	25 (29.8%)	8 (9.2%)	16 (20.5%)	8 (9.5%)	57 (17.1%)
No	59 (70.2%)	79 (90.8%)	62 (79.5%)	76 (90.5%)	276 (82.8%)
**Household characteristics**	**Uttara**	**Mirpur**	**Mugda**	**Chawkbazaar**	**Total**
	**(N = 85)**	**(N = 81)**	**(N = 65)**	**(N = 66)**	**(N = 297)**
**Number of people living in the house**					
Median [Min, Max]	4.00 [3.00, 7.00]	4.00 [3.00, 13.0]	4.00 [2.00, 11.0]	5.00 [3.00, 11.0]	4.00 [2.00, 13.0]
**Moved in the last 5 years**					
Yes	24 (28.2%)	6 (7.4%)	33 (50.8%)	26 (39.4%)	89 (30.0%)
No	61 (71.8%)	75 (92.6%)	32 (49.2%)	39 (59.1%)	207 (69.7%)
Don’t know	0 (0%)	0 (0%)	0 (0%)	1 (1.5%)	1 (0.3%)
**Type of dwelling**					
Independent	**11 (12.9%)**	1 (1.2%)	6 (9.2%)	0 (0%)	18 (6.1%)
Multi-storied	9 (10.6%)	0 (0%)	48 (73.8%)	66 (100%)	123 (41.4%)
Semi-pucca	65 (76.5%)	19 (23.5%)	10 (15.4%)	0 (0%)	94 (31.6%)
Informal settlement	0 (0%)	61 (75.3%)	1 (1.5%)	0 (0%)	62 (20.9%)
**Household Income Index**					
Low	36 (42.4%)	68 (84.0%)	9 (13.8%)	3 (4.5%)	116 (39.1%)
Moderate	32 (37.6%)	13 (16.0%)	24 (36.9%)	16 (24.2%)	85 (28.6%)
High	17 (20.0%)	0 (0%)	32 (49.2%)	47 (71.2%)	96 (32.3%)
**Social Capital Index**					
Low/moderate	47 (56.0%)	38 (43.7%)	38 (48.7%)	39 (46.4%)	162 (48.6%)
High	37 (44.0%)	49 (56.3%)	40 (51.3%)	45 (53.6%)	171 (51.4%)

*Religious education.

Parent participants had a median age of 32.5, with a higher median in Mirpur (35.2). 83.5% of the participants were women. Overall, 54.2% of the participants had a primary school education or less, and only 3% held a university degree, primarily from Mugda and Chawkbazaar. Among household members, Uttara and Mugda had the highest percentages (27.1% and 21.5%, respectively) of at least one reported episode in 2023. Mugda and Chawkbazaar reported the highest percentage of lifetime episodes of dengue with 36.9% and 25.8%, respectively, compared to the other settings (Table 1). The median age of the children was 6.7 years, with almost 60% attending school or daycare.

Of the 412 children, only 333 blood samples were taken (79 parents opted out of participating in the RDT; their characteristics are presented in Table S2.3 in S2 Material). 57 samples were positive for recent or previous infection (S2 Material). The crude seroprevalence for any infection was 17%. The adjusted seroprevalence of dengue infection was 14%, while the seroprevalence for recent or acute infection (IgM) was 5% and for previous exposure (IgG) was 3% (S2 Material). Uttara and Mugda had the highest percentages of seropositivity for dengue infections, with 29.8% and 20.5%, respectively. Of the 12 children reported by parents to have had a dengue episode in 2023, only two children were confirmed to be positive by the test.

### Knowledge, attitudes, and practice

In terms of dengue knowledge, 68.0% recognized that dengue is a disease transmitted by mosquitoes, while 94.3% considered fever to be the main symptom of infection, followed by nausea or vomiting (53.2%). Regarding sources of information about dengue, 74.4% of participants stated that television was a primary source, followed by friends and family (45.6%). Regarding attitudes, 77.4% of parents believed that dengue was a problem for themselves and their families. Only 33.7% of participants felt at risk of contracting dengue, whereas 43.4% believed that the community could prevent dengue. Regarding practices, the vast majority of respondents (94.6%) will go to the hospital if they suspect dengue. Among the measures practiced for controlling dengue, 43.7% fumigated their homes, while 31.3% cleaned stagnant water reservoirs. Finally, 95.6% indicated that the city corporation implemented mosquito control measures in 2023 in their community. Participants reported that the actions taken by the city corporation were: 30.6% fumigation inside the home, 92.3% fumigation outside, and 7.1% providing recommendations to prevent or control dengue. The frequency and percentage of responses to each of the KAP questions are provided in S3 Material.

The average knowledge, attitudes, and practice scores differed across the four study sites, with practice scores higher in Uttara and Mirpur (85.7% and 84.5%, respectively) than in Mugda and Chawkbazaar (67.0% and 63.6%, respectively). The categorization of domain scores into three levels revealed that participants in Uttara and Mirpur had higher positive attitudes towards dengue prevention (75.3% and 80.2%, respectively) and towards extensive preventive practices (88.2% and 85.2%, respectively) than those in Mugda and Chawkbazaar. In all four settings, most participants (over 70%) had knowledge levels above the essential level. A total of 187 (63%) participants had a moderate total KAP score. Only 90 participants had a KAP score higher than 20.25 points (Table 2).

**Table 2:** Categories of KAP by domain and total.

KAP total and domain-level scores	Uttara	Mirpur	Mugda	Chawkbazaar	Total
	(N = 85)	(N = 81)	(N = 65)	(N = 66)	(N = 297)
**Knowledge (score 14)**					
Essential (<7)	18 (21.2%)	12 (14.8%)	10 (15.4%)	17 (25.8%)	57 (19.2%)
Intermediate (7 -10.5)	50 (58.8%)	43 (53.1%)	45 (69.2%)	43 (65.2%)	181 (60.9%)
Knowledgeable (≥ 10.5)	17 (20.0%)	26 (32.1%)	10 (15.4%)	6 (9.1%)	59 (19.9%)
**Attitude (score 6)**					
Negative (<3)	2 (2.4%)	2 (2.5%)	7 (10.8%)	1 (1.5%)	12 (4.0%)
Neutral (3 - 4.5)	19 (22.4%)	14 (17.3%)	27 (41.5%)	34 (51.5%)	94 (31.6%)
Positive (≥ 4.5)	64 (75.3%)	65 (80.2%)	31 (47.7%)	31 (47.0%)	191 (64.3%)
**Practice (score 7)**					
Introductory (<3.5)	2 (2.4%)	0 (0%)	12 (18.5%)	10 (15.2%)	24 (8.1%)
Partial (3.5-5.25)	8 (9.4%)	12 (14.8%)	32 (49.2%)	37 (56.1%)	89 (30.0%)
Extensive (≥ 5.25)	75 (88.2%)	69 (85.2%)	21 (32.3%)	19 (28.8%)	184 (62.0%)
**KAP (total 27)**					
Low (<13.5)	2 (2.4%)	1 (1.2%)	8 (12.3%)	9 (13.6%)	21 (6.7%)
Moderate (13.5-20.25)	50 (58.8%)	43 (53.1%)	48 (73.8%)	45 (69.7%)	187 (63.0%)
High (≥ 20.25)	33 (38.8%)	37 (45.7%)	9 (13.8%)	11 (16.7%)	90 (30.3%)

The Kendall’s tau test of KAP domains revealed a significant but weak positive correlation between knowledge and attitudes, as well as between knowledge and practices. On the other hand, attitudes and practices show a moderate positive correlation (Table 3).

**Table 3:** Kendall’s rank correlation.

Domains	tau	p-value
Knowledge vs Attitude	0.12	0.02
Knowledge vs Practice	0.13	0.01
Attitude vs Practice	0.32	<0.001

### Association between children’s seropositivity and parents’ KAP

In the univariate analyses between parents’ KAP and child seropositivity, attitudes had an RR of 0.82 (95% CI 0.67-1.01) (S4 Material). In the multivariable regression model, the magnitude and direction of the associations were similar; the attitude score (a continuous variable) provided a better fit and was retained for the adjusted analyses. In this analysis, most predictors were not significantly associated with the outcome. Three variables (child’s school attendance, the parent’s working status, and hospitalization of a family member for dengue) had p-values > 0.9. These variables were nevertheless included in the adjusted regression models due to their theoretical relevance and potential for confounding. In the multivariable regression model, a parent’s higher attitude score was associated with a decreased child risk of dengue (aRR, 0.77; 95% CI, 0.60-0.99) (Table 4).

**Table 4:** Association of parents’ KAP with seropositivity in children.

Variable	aRR
**Attitude Score**	0.77 (0.60-0.99)
**Child’s previous dengue**	
No	Ref.
Yes	0.74 (0.15-3.78)
**Child age in number**	1.08 (0.97-1.20)
**Child attends school**	
No	Ref.
Yes	1.01 (0.57-1.78)
**Chronic disease of the child**	
No	Ref.
Yes	1.55 (0.44-5.50)
**Child’s gender**	
Male	Ref.
Female	0.91 (0.54-1.56)
**Parent’s age**	0.98 (0.94-1.02)
**Parent’s gender**	
Male	Ref.
Female	0.69 (0.37-1.29)
**Level of education**	
Less than primary school	Ref.
Secondary or more	0.96 (0.56-1.65)
**Working status**	
Not working at the moment	Ref.
Working	0.91 (0.35-2.35
**Hospitalization of a family member**	
No	Ref.
Yes	0.97 (0.31-3.07)
**Change of residency in the last 5 years**	
No	Ref.
Yes	0.62 (0.33-1.16)
**Number of people with previous dengue in the household**	1.52 (0.84-2.75)
**Household index**	
Low	Ref.
Medium	0.83 (0.45-1.54)
High	0.79 (0.40-1.53)
**Water storage**	
No storage or closed	Ref.
Presence of open water storage	0.78 (0.31-1.97)
**Social Capital**	
Low/Moderate	1
High	0.75 (0.45-1.26)

## Discussion

Understanding the association between parents’ KAP and dengue seropositivity in emerging settings, such as Dhaka, particularly among vulnerable groups, like children, is crucial for improving control and prevention strategies. In this first study on the association between parents’ KAP and dengue seropositivity in children during the 2023 epidemic, it was found that seroprevalence differed across neighborhoods and that parents did not report the vast majority of participants’ dengue infections for that year. Intermediate levels of knowledge were reported across all study neighborhoods, while attitude and practice scores were higher in Uttara and Mirpur. We also found that KAP domains were not strongly correlated with one another. Only attitudes were significantly associated with dengue seropositivity, whereas typical sociodemographic and other dengue risk indicators (e.g., child age, parent gender, working status, and water storage) were not.

Crude seroprevalence results for recent and previous infections in our study showed lower rates than those reported by the country during the 2023 dengue outbreak (15% in children aged 15 years or less) [6]. Other studies in Bangladesh reported a seroprevalence of 54.9% for IgG in children in the general population in 2017 and 42.6% for IgM in hospitalized children in 2023 [27,28]. Our result is also low compared to other countries, such as Brazil and Colombia, where seroprevalence was 25.1% and 87.9% in children for dengue infection [25,54]. However, it is essential to consider that our test was taken by participants without fever, the main symptom of dengue, which is the indication for performing a rapid test according to local authorities [78]. Also, the reported independent studies used ELISA-based methods, from single assays [27,54], to multi-assay confirmatory algorithms [25], or the SD Bioline test [28]. Regarding asymptomatic dengue seroprevalence, our study included children aged 2–8 years, who were found to have higher asymptomatic seroprevalence in Thailand [26]. Additionally, a systematic review revealed that the seroprevalence of asymptomatic dengue varies significantly, with results ranging from 0% to 97% [79]. Thus, a lower seroprevalence among asymptomatic patients is expected and suggests ongoing transmission and potential future outbreaks. Regarding the gender of the children, others have found higher seroprevalence among male adolescents and adults due to more outdoor time [54,80]. However, there were no statistically significant differences between genders in our study. This can be explained by similar exposure to dengue outside the home, driven by the study population’s similar school attendance between boys and girls.

In terms of KAP, the results showed notable differences from previous studies in the country [36,37,65,81–83]. The intermediate level of the knowledge score in our study was consistent with findings from two other studies conducted in Bangladesh, which reported that 66% to 68% of participants also had intermediate knowledge scores [36,81]. Other studies have shown that higher knowledge levels are achieved after receiving information or with higher levels of education [36,37]. Regarding specific aspects of knowledge, we found that 68% of participants recognized the mosquito as a mode of transmission, a number that is lower than that reported in another study (80%) [66]. Regarding attitudes, we found that participants with lower levels of education and those with at least one family member hospitalized for dengue had more positive attitudes towards dengue prevention and control. Previous studies in Dhaka found that lower attitude scores were associated with lower education levels, unemployment, and not being exposed to dengue, whereas negative attitudes were associated with perceived financial loss or risk of contracting the disease [36,37,82,83]. In terms of practices, the overall population in our study presented high scores (62.0%). With respect to specific practices, only 18.2% of participants cover the water containers. This practice of controlling mosquito breeding sites has been reported in Bangladesh, with percentages ranging from 53.6% to 98% [65,81]. Our result may be due to the timing of the questionnaire in November-December, which coincided with the end of the dengue season. Our study found that a higher percentage of participants reported consulting the hospital when they suspected dengue than had been reported previously [65].

Factors such as the complexity of the KAP approach and the cultural context of Dhaka can explain our results. The complex interconnections among questions within and between domains cannot be clearly mapped using the standard correlation measures. In this regard, several studies have demonstrated variability in the correlation between domains. In studies analyzing KAP about dengue, the methods of choice for assessing correlations between domains were Spearman’s or Kendall’s Tau [54,65,82,84]. Although the results generally showed a significant correlation between the domains, the strength of knowledge and practices may vary (R value of 0.25 or 0.35), as well as with attitudes (0.27 or 0.30), in studies conducted in the same country [65,82]. Our study employed a questionnaire previously used in Brazil, for which correlations with Kendall’s Tau were significant, but lower between attitudes and practices (Tau = 0.12, p = 0.008) compared to our study [54]. This demonstrates the effect of adapting the questionnaire content to the country’s language and culture, as well as the possible influence of context on these correlations. Thus, the integration independence analysis using copulas can help capture the complexity of the non-linear relationships in KAP questionnaires [85].

This study found that, in the four neighborhoods studied in Dhaka, factors determining children’s seropositivity were related to attitudes towards the disease. In this regard, other studies on the association of seropositivity with dengue found no single determinant. A study in Brazil has found that households with higher KAP scores have a higher relative risk of dengue seropositivity in children [54], while in Malaysia, low knowledge, attitude, and practice scores were associated with high seropositivity in adults [64]. Another Malaysian study reported that low knowledge scores were associated with lower seropositivity, while prevention practices were not associated with dengue seropositivity [63]. While our study did not find an association with other factors, the literature reports that a child older than 8 years [25], a family member with dengue in the same season, the presence of more than one uncovered tank [27], and community interventions [86] were associated with dengue seroprevalence. These findings demonstrate that adult behavioral factors, not only demographic, physiological, and clinical factors, may be related to the risk of infection in children and warrant further exploration.

Ultimately, the local context is crucial for interpreting KAP evaluation results. Bangladesh has faced dengue epidemics since 2019, and in 2023, it recorded the highest number of cases in its history [9,43,44]. Dhaka, as a densely populated megacity with developing infrastructure, faces significant inequalities in access to health and sanitation services [87,88]. In this scenario, control measures such as fumigation and awareness campaigns are implemented annually [8,89]. As a result, the disease knowledge and control practices are familiar to the population, but the correlation between them is weak. This is consistent with observations in other contexts where practices were not directly associated with knowledge [35,66]. This can also be explained by the TPB, on which the questionnaire was based. The three main constructs of the TPB (attitude toward the behavior, subjective norm, and perceived behavioral control) are independent, and their relative weight in determining behavioral intention is expected to vary across behaviors and populations [55]. The questionnaire in this study focused on attitudes toward risk perception and the possibility of prevention; these two attitudes can influence childhood seropositivity. It has been reported that perceiving children as at risk motivates the adoption of protective measures [90,91].

Our KAP findings suggest that the communities possessed knowledge about dengue and relevant prevention and control practices, which could serve as a foundation for more participatory, community-based control interventions. Individuals with higher scores may be well-positioned to serve as community advocates in prevention initiatives, raising awareness among neighbors [92,93]. This peer-based community engagement approach could be a viable strategy to explore, alongside the country’s current surveillance and information initiatives. At the policy level, incorporating routine assessment of risk perception into dengue surveillance could help more effectively target risk communication to the communities where it is most needed.

Our study had some limitations related to potential bias and to the generalizability of the findings. Regarding selection bias, the sampling approach may have introduced bias as households farther from the neighborhood center had a lower probability of being selected to participate. Additionally, participants who agreed to take part in the study may have been more interested in or more aware of dengue and may have differed systematically from non-participant households. Nevertheless, the similarity between the demographic characteristics of the participants, such as age, sex, and number of children in the household, and the vital statistics reported by the government suggest that the potential for selection bias related to demographic factors may be limited. Information bias may also be present, as the questionnaire items were self-reported, and social desirability bias may have been present. Although the questionnaire underwent content validity assessment for the local context prior to implementation, the post-implementation Cronbach’s alpha was lower than recommended for a questionnaire, indicating potential measurement error that was not correctable at the design stage. Also, the use of a rapid test provides a practical screening tool but not as a diagnostic measure, and its estimates of previous dengue infection may not be directly comparable to laboratory-based serological methods such as ELISA [60,61]. Although the manufacturer reports sensitivity above 90% for both IgG and IgM [62], these studies report values as low as 50.73% for IgM and 69.03% for IgG [60,61]. This may have led to some participants being misclassified as negative and the true seroprevalence in this population may be higher than observed [61]. Regarding generalizability, the sample was drawn from urban locations based on accessibility and high incidence rates across two city corporations that may differ in vector control strategies and governance structures; thus, the findings may not be extended to rural areas or to cities with different governance structures. This study was conducted at the end of the 2023 dengue season without serotype data, therefore the findings should be interpreted with the specific transmission and population immunity context of this period. In terms of sample size, this was appropriate for a pilot study intended to inform a cRCT; however, the observed associations should be considered preliminary, and confirmation in a larger study employing a mixed-effects regression approach is warranted. Furthermore, since this is a cross-sectional design, causal relationships among the factors cannot be established; the observed results reflect the strength and direction of association, not causal effects. Finally, residual confounding, as well as confounding from unmeasured variables not collected, cannot be completely ruled out despite the inclusion of relevant variables in the regression models.

## Conclusion

The results of our study demonstrate the need to thoroughly examine KAP by systematically constructing the KAP score and applying it in univariable models. Additionally, these diverse results indicate that seropositivity is associated with factors that may vary by context and population. These findings suggest an association between parental perceptions, and opinions, and prevention activities, which may in turn be relevant to understanding disease prevalence in children in this population. A contextualization of the factors through a dialogue with the community is required to understand these results in depth and to generate prevention and control activities adapted to the community.

## Supporting information

S1 Material Questionnaire, KAP scoring, household index, and social capital index. (PDF)

S2 Material Seroprevalence and children’s characteristics by neighborhood. Characteristics of the children who did not take part in the serology test.
(PDF)

S3 Material KAP detailed descriptive tables. (PDF)

S4 Material Correlation and additional regression models. (PDF)

S1 Checklist STROBE Checklist and CHECKAP List. Completed STROBE checklist for cross-sectional studies. The checklist is from the STROBE statement (von Elm E, Altman DG, Egger M, Pocock SJ, Gotzsche PC, Vandenbroucke JP. The Strengthening the Reporting of Observational Studies in Epidemiology (STROBE) Statement: guidelines for reporting observational studies. PLoS Med 4(10): e296. https://doi.org/10.1371/journal.pmed.0040296), available at https://www.strobe-statement.org/checklists/, and is distributed under the Creative Commons Attribution (CC BY 4.0) license (https://creativecommons.org/licenses/by/4.0/). ** Zarei, F., Dehghani, A., Ratansiri, A., Ghaffari, M., Raina, S., Halimi, A., Rakhshanderou, S., Isamel, S., Amiri, P., Aminafshar, A., & Mosavi Jarrahi, A. (2024). ChecKAP: A Checklist for Reporting a Knowledge, Attitude, and Practice (KAP) Study. Asian Pacific Journal of Cancer Prevention, 25(7), 2573–2577. https://doi.org/10.31557/APJCP.2024.25.7.2573.
(PDF)
