# Supplementary material for: Cross-sectional study on the association between seroprevalence among children and their parents’ knowledge, attitudes, and practices in a dengue-endemic city in Bangladesh
Source: PLoS Negl Trop Dis. 2026 Oct 21;20(10):e0014802. doi: 10.1371/journal.pntd.0014802 (PMC13712692; doi:10.1371/journal.pntd.0014802)
Supplement: Supplementary file 1 [file pntd.0014802.s001.pdf]

Supplement 1
Questionnaire, KAP score. Household index and Social Capital index

Cross-sectional study on the association between seroprevalence among children and their parents' knowledge, attitudes, and practices in a dengue-endemic city in Bangladesh

1.1 KAP Questionnaire

Study title: A formative study on the acceptability of dengue antibody testing among children aged 2-12 years and community-based interventions for dengue control in Dhaka, Bangladesh

গবেষণার শিরোনাম: ২-১২ বছর বয়সী শিশুদের মধ্যে ডেঙ্গু অ্যান্টিবডি পরীক্ষার গ্রহণযোগ্যতা এবং ঢাকা, বাংলাদেশে ডেঙ্গু নিয়ন্ত্রণের জন্য এলাকা-ভিত্তিক ইন্টারভেনশনের উপর একটি প্রাথমিক গবেষণা

Study site:<br>গবেষণা ক্ষেত্রঃ	Cluster number:<br>ক্লাস্টার নাম্বারঃ	Date (dd/mm/yy): _ _ / _ _ / _ _<br>তারিখ (দিন/মাস/বছর)ঃ
Arrival time: _ _ H _ _ Min<br>পৌঁছুনর সময়ঃ ___ ঘন্টা ___ মিনিট	Departure time: _ _ H _ _ Min<br>শেষ করার সময়ঃ ___ ঘন্টা ___ মিনিট	
Household ID:<br>খানার আইডিঃ	Individual ID:<br>ব্যাক্তি আইডিঃ	Team Research ID:<br>গবেষণা দল আইডিঃ
Address:<br>ঠিকানাঃ		
Visit: O Baseline O Follow-up<br>সময়ঃ O বেসলাইন O ফলোআপ		

Section 1: Eligibility – asked only at enrollment<br>সেকশন ১ঃ অংশগ্রহণের যোগ্যতাঃ শুধুমাত্র তালিকাভুক্তির সময় প্রযোজ্য		
E1.	Are you one of the principal adults (male or female) of the household?<br><br>Definition: One of the primary adults for managing household affairs (functional and/or financial aspects)<br>আপনি কি এই খানার প্রধান ব্যাক্তিদের একজন?	• Yes (হ্যাঁ)<br>• No
E2.	Are you able to answer questions about household income, sanitation services, household illnesses, and water storage practices in the household?	• Yes (হ্যাঁ)<br>• No
E3.	Are you and your family planning to move from this address in the next 6 months?<br>আপনি এবং আপনার পরিবার কি পরবর্তী ৬ মাসের মাঝে এই ঠিকানা থেকে চলে যাবার কোন পরিকল্পনা করছেন?	• Yes (হ্যাঁ)<br>• No (না)<br>• Don't know (জানিনা)
E4.	Are you the parent or legal guardian of a child between the age of 2 years to 12 years old that also lives in the household?<br>এই খানা তে কি ২ বছর থেকে ১২ বছর বয়সী কোন বাচ্চা আছে?	• Yes (হ্যাঁ)<br>• No (না)
E5.	How many of your children between the ages of 2 years to 12 years old live in this household?<br>২ বছর থেকে ১২ বছর বয়সী কয়টি বাচ্চা এই খানা তে আছে?	__ number<br>______ নাম্বার
E6.	Do any of the children suffer from excessive bleeding (hemophilia)?	__ number<br>______ নাম্বার<br>(if E5 = E4, end questionnaire)

	বাচ্চাদের কেউ কি অতিরিক্ত রক্তক্ষরণে ভুগছে?<br><br>These children will be excluded from participating.	(যদি E5 = E4 হয়, তাহলে প্রশ্নপত্র শেষ করুন)
E7.	Are any of the children currently suffering from fever?<br><br>These children will be excluded from participating.	__ number<br>______ নাম্বার<br>(if E5 = E4, end questionnaire)<br>(যদি E5 = E4 হয়, তাহলে প্রশ্নপত্র শেষ করুন)
Section 2: Socio-economic information (SE) – asked only at baseline<br>সেকশন ২ঃ আর্থসামাজিক তথ্যাবলী - শুধু বেসলাইনে প্রশ্ন করতে হবে		
SE1.	When were you born (please give the month and year)?<br>আপনার গত জন্মদিনে আপনার বয়স কত ছিল?	__MM<br>____YYYY<br>____ বছর
SE2.	What is your gender?<br>আপনার লিঙ্গ কোনটি?	• Male (পুরুষ)<br>• Female (মহিলা)<br>• Third gender (হিজরা)<br>• Other (অন্যান্য)<br>• Does not want to answer (বলতে চাচ্ছি না)
SE3.	Can you read and write?<br>আপনি কি পড়তে ও লিখতে পারেন?	• Can read and write (পড়তে ও লিখতে পারেন)<br>• Can only read (শুধু পড়তে পারেন)<br>• Can't read and write (পড়তে ও লিখতে পারেন না)
SE4.	What is the highest level of education that you have completed ?<br>আপনি সর্বোচ্চ কোন শ্রেণি পাস করেছেন?	1 = Grade 1<br>2 = Grade 2<br>3 = Grade 3<br>4 = Grade 4<br>5 = Grade 5<br>6 = Grade 6<br>7 = Grade 7<br>8 = Grade 8<br>9 = Grade 9<br>10 = Grade 10<br>11 = SSC/Dakhil<br>12 = HSC/Alim<br>13 = Diploma/Vocational<br>14 = BA/BSC/BCom/Fazil/ Graduate/BA (Hons)<br>15 = MA/Ph.D./Kamil<br>16 = Hafezi/ Qawmi/ Kharizi<br>17 = Did not complete the 1st grade<br>88 = Refused
SE5.	What is your working status?<br>আপনি কি এখন কোন কাজ করছেন?	• Working currently (বর্তমানে কাজ করছি)<br>• Household work/housework (skip to SE10) (বাড়ির কাজ করছি শুধু - SE10 এ চলে যান)

		• Looking for work (skip to SE10) (কাজ খুঁজছি - SE10 এ চলে যান)<br>• Does not work (skip to SE10) (কাজ করছি না - SE10 এ চলে যান)
SE6.	If working, what is the field of employment?<br>এখন কোন কাজে নিযুক্ত থাকলে, কোন ধরনের কাজে নিযুক্ত আছেন?	• Housewife = 01 (গৃহিণী)<br>• Farmer at own field (Agriculture) = 02 (নিজের জমিতে কাজ করা কৃষক)<br>• Farmer at other's field on lease (Agriculture)= 03 (অন্যের জমিতে কাজ করা কৃষক)<br>• Day labourer (Other)= 04 (দিনমজুর)<br>• Skilled labourer= 05 (প্রশিক্ষিত মজুর)<br>• Rickshaw/van/cart puller/baby taxi driver/boatman= 06 (রিকশা/ভ্যান/বেবি ট্যাক্সি/নৌকা চালক)<br>• Fisherman= 07 (জেলে)<br>• Salaried worker= 08 (বেতনভুক কর্মচারি)<br>• Businessman = 09 (ব্যবসায়ী)<br>• Household help= 10 (গৃহকর্মী)<br>• Doctor/engineer= 11 (ডাক্তার/প্রকৌশলী)<br>• Homemaker= 12 (গৃহকর্মী)<br>• Retired= 13 (অবসরপ্রাপ্ত)<br>• Student= 14 (ছাত্র)<br>• Teacher = 15 (শিক্ষক)<br>• Other = 96 (অন্যান্য)<br>• If "96", please specify: [_________]<br>• (নির্দিষ্ট করুন)
SE7.	How many people live in the household?<br>এই খানায় কতজন মানুষ বাস করে?<br><br>[household defined as where several people live, cook, and eat together. Include adults, children, infants, possible participants]<br>[খানা বলতে যেখানে একের অধিক মানুষ একসাথে থাকা, রান্না এবং খাওয়ার কাজ করেন বোঝানো হয়েছে]	__ people<br>_____ মানুষ
Section 3: Dwelling characteristics and environment situation (DE) – to be asked only at baseline<br>সেকশন ৩: বাসস্থান বৈশিষ্ট্য এবং পরিবেশ পরিস্থিতি (DE) - শুধুমাত্র বেসলাইনে জিজ্ঞাসা করা হবে		
DE1.	Have you moved in the past 5 years?	• Yes<br>• হ্যাঁ<br>• No (skip to DE3.)<br>• না<br>• Does not know (skip to DE3.)<br>• জানিনা

DE2.	What were the top three reasons for moving?<br>বাসস্থান পরিবর্তন করার প্রধান তিনটি কারণ কি ছিল?	• Financial reasons<br>• অর্থনৈতিক কারণ<br>• Work related reasons<br>• কাজ সংক্রান্ত কারণ<br>• Family related reasons<br>• পরিবার সংশ্লিষ্ট কারণ<br>• Neighbourhood insecurity<br>• প্রতিবেশী সংক্রান্ত নিরাপত্তাহীনতা<br>• Slum evictions<br>• Community services<br>• কমিউনিটির সেবা<br>• Extreme heat<br>• মাত্রাতিরিক্ত গরমের কারণে<br>• Flooding<br>• বন্যা<br>• Other: specify<br>• অন্যান্যঃ নির্দিষ্ট করে বলুন<br>• Does not know<br>• জানিনা<br>• Does not want to answer<br>• উত্তর প্রদানে অনিচ্ছুক
DE3.	What type of dwelling is this?<br>আপনার বাসস্থানটি কেমন?	• Independent<br>• Multi-storied<br>• Semi-pucca<br>• Slum<br>• বস্তি<br>• Floating/Rootless<br>• ভাসমান বা শিকড়হীন<br>• None of the above<br>• উপরের কোনটি নয়
DE4.	What is the main material of the floor?<br>ঝের প্রধান উপাদান কি?	• Cement<br>• সিমেন্ট<br>• Earth / sand<br>• মাটি/ বালু<br>• Other material<br>• অন্যান্য উপাদান
DE5.	What is the main material of the exterior walls?<br>বাইরের দেয়ালের প্রধান উপাদান কি?	• Cement<br>• সিমেন্ট<br>• Other material<br>• অন্যান্য উপাদান
DE6.	What is the main material of the roof?<br>ছাদের প্রধান উপাদান কি?	• Cement<br>• সিমেন্ট<br>• Other material<br>• অন্যান্য উপাদান
DE7.	Does your household have a television?<br>আপনার বাসায় কি টেলিভিশন রয়েছে?	• Yes<br>• হ্যাঁ

		• No<br>• না
DE8.	Does your household have a refrigerator?<br>আপনার বাসায় কি ফ্রিজ রয়েছে?	• Yes<br>• হ্যাঁ<br>• No<br>• না
DE9.	Does your household have an almirah/wardrobe?<br>আপনার বাসায় কি আলমারি বা ওয়ারড্রব রয়েছে?	• Yes<br>• হ্যাঁ<br>• No<br>• না
DE10.	Does your household have an electric fan?<br>আপনার বাসায় কি একটি বৈদ্যুতিক পাখা রয়েছে?	• Yes<br>• হ্যাঁ<br>• No<br>• না
DE11.	How many rooms does the dwelling have?<br>আপনার বাসায় কতটি রুম রয়েছে?	• __ rooms<br>• – রুম<br>• Does not know<br>• জানিনা<br>• Does not want to answer<br>• উত্তর দিতে অনিচ্ছুক
DE12.	Does this dwelling have a backyard or any external areas?<br>এই বাসস্থানের কি বাড়ির পিছনের দিকের উঠোন বা বাইরের কোন এলাকা আছে?<br><br>*This space could be used for any purpose, and will be assessed as part of the entomological (mosquito) assessment.*<br>*এই স্থানটি যেকোন উদ্দেশ্যে ব্যবহার করা হতে পারে এবং কীটতত্ত্ববিষয়ক (মশা) মূল্যায়নের অংশ হিসাবে মূল্যায়ন করা হবে।*	• Yes<br>• হ্যাঁ<br>• No<br>• না<br>• Does not know<br>• জানিনা<br>• Does not want to answer<br>• উত্তর দিতে অনিচ্ছুক
DE13.	The garbage in this household is:<br>এই বাসার আবর্জনা	• Collected directly by a cleaning service (public or private)<br>• সরাসরি পরিচ্ছন্নতা সেবা দ্বারা সংগৃহীত (সরকারি বা প্রাইভেট)<br>• Collected from a cleaning service container (public or private)<br>• পরিচ্ছন্নতা সেবার কন্টেইনার থেকে সংগৃহীত (সরকারি বা প্রাইভেট)<br>• Burned (within the property) (skip to DE15)<br>• পুড়িয়ে ফেলা হয় (বাসার এলাকার ভেতরে) (DE15 পরিহার করুন)<br>• Buried (within the property) (skip to DE15)

		● পুঁতে ফেলা হয় (বাসার এলাকার ভেতরে) (DE15 পরিহার করুন)<br>● Thrown on a wasteland or on a public place (skip to DE15)<br>● ময়লাস্তুপে বা পাবলিক স্থানে নিক্ষেপ করা হয় (DE15 এ যান)<br>● Thrown on a river, lake or on the sea (skip to DE15)<br>● নদী, লেক বা সমুদ্রে নিক্ষেপ করা হয় (DE15 এ যান)<br>● Other: specify (skip to DE15)<br>● অন্যান্যঃ নির্দিষ্ট করুন (DE15 এ যান)<br>● Does not know (skip to DE15)<br>● জানিনা (DE15 এ যান)<br>● Does not want to answer (skip to DE15)<br>● উত্তর প্রদানে অনিচ্ছুক (DE15 এ যান)
DE14.	If collected, how often is the garbage collected?<br>যদি সংগ্রহ করা হয়, কত ঘন ঘন সময়ে ময়লা সংগ্রহ করা হয়?	● Daily<br>● প্রতিদিন<br>● 3 times/week<br>● সপ্তাহে তিন দিন<br>● Weekly<br>● প্রতি সপ্তাহে ১ বার<br>● Each 2 weeks<br>● প্রতি ২ সপ্তাহে এক বার<br>● Monthly<br>● মাসিকভাবে<br>● Sporadically (less than monthly)<br>● বিক্ষিপ্তভাবে<br>● Other: specify<br>● অন্যান্যঃ নির্দিষ্ট করুন<br>● Does not know<br>● জানিনা<br>● Does not want to answer<br>● উত্তর প্রদানে অনিচ্ছুক
DE15.	What are the toilet facilities at your dwelling?<br><br>আপনার বাসায় টয়লেটের ব্যবস্থা কেমন?<br><br>*Allow multiple answers*<br>*একাধিক উত্তর দিতে পারবেন*	● Safe drainage by flushing/pouring water<br>● ফ্লাশ বা পানি ঢেলে নিরাপদ নিষ্কাশন ব্যবস্থা<br>● Unsafe drainage by flushing/pouring water<br>● ফ্লাশ বা পানি ঢেলে অনিরাপদ নিষ্কাশন ব্যবস্থা<br>● Pit latrine with slab/ventilated improved latrine/composting latrine<br>● স্ল্যাব যুক্ত পিট টয়লেট, ভ্যান্টিলেটরযুক্ত টয়লেট, কমপোস্টিং টয়লেট<br>● Pit latrine without slab/open pit

		• স্ল্যাব বিহীন পিট টয়লেট/ খোলা পিট টয়লেট<br>• Raw/open/hanging latrine (permanent/temporary)<br>• খোলা বা ঝুলন্ত টয়লেট (স্থায়ী বা অস্থায়ী)<br>• Open defecation/no latrine available (skip to DE19)<br>• খোলা জায়গায় মলত্যাগ/ টয়লেট নেই (DE19 এ যান)
DE16.	What are your sources of water for drinking and household use?<br>বাসায় ব্যবহারের জন্য এবং খাওয়ার জন্য আপনার পানির উৎস কি?	• Taped/Piped (Supply)<br>• টেপ/ পাইপ (সরবরাহ)<br>• Tubewell (deep/shallow)<br>• নলকূপ (গভীর, অগভীর)<br>• Bottled water/water jar<br>• পানির বোতল/ পানির বয়াম<br>• Well<br>• কূপ<br>• Pond/river/lake<br>• পুকুর/নদী/লেক<br>• Spring<br>• ঝর্ণা<br>• Rain water<br>• বৃষ্টির পানি<br>• Other: specify<br>• অন্যান্যঃ নির্দিষ্ট
DE17.	How often, on average, does your home lack water?<br>সাধারনত, গড়ে, কত ঘন ঘন আপনার বাসায় পানির স্বল্পতা দেখা যায়?	• Never (skip to DE219<br>• কখনোই না (DE19 এ যান)<br>• Sporadically (few times during the year, less than 6 times/year)<br>• মাঝেমধ্যে (বছরে কয়েকবার, বছরে ৬ বারের কম)<br>• Almost every month<br>• প্রায় প্রতি মাসে<br>• Almost every week<br>• প্রায় প্রতি সপ্তাহে<br>• Does not know (skip to DE19)<br>• জানিনা (DE19 এ যান)<br>• Does not want to answer (skip to DE19)<br>• উত্তর প্রদানে অনিচ্ছুক (DE19 এ যান)
DE18.	Does this change over the year (between dry and wet seasons)?<br>বছরের বিভিন্ন সময়ে কি এর মাঝে কোন পরিবর্তন লক্ষ্যিত হয় (শুষ্ক এবং আর্দ্র ঋতুর মধ্যে)?	• No<br>• না<br>• Yes, less water during the dry season<br>• হ্যাঁ, শুষ্ক মৌসুমে পানির স্বল্পতা থাকে<br>• Yes, less water during the wet season<br>• হ্যাঁ, আর্দ্র ঋতুর সময় পানির স্বল্পতা থাকে<br>• Other: specify

		• অন্যান্যঃ নির্দিষ্ট<br>• Does not know<br>• জানিনা<br>• Does not want to answer<br>• উত্তর প্রদানে অনিচ্ছুক
DE19.	Does this residence have an individual water tank?<br>এই বাসায় কি ব্যক্তিগত পানির ট্যাংক রয়েছে?	• Yes<br>• হ্যাঁ<br>• No (skip to DE21)<br>• না (DE21 এ যান)<br>• Does not know (skip to DE21)<br>• জানিনা (DE21 এ যান)<br>• Does not want to answer (skip to DE21)<br>• উত্তর প্রদানে অনিচ্ছুক (DE21 এ যান)
DE20.	If yes, is it:<br>যদি হ্যাঁ হয়, তাহলে সেটা কি;	• Open<br>• খোলা<br>• Covered<br>• আবৃত<br>• Covered and sealed<br>• আবৃত এবং বদ্ধ<br>• Does not know<br>• জানিনা<br>• Does not want to answer<br>• উত্তর প্রদানে অনিচ্ছুক
DE21.	Do you store water in other ways?<br>বাসায় কি পানি সংগ্রহে রাখার অন্য কোন ব্যবস্থা রয়েছে?	• No, I do not<br>• না, আমি এমন কোন ব্যবস্থা নেই<br>• Closed containers inside the home<br>• Open containers inside the home<br>• Closed containers outside the home<br>• Open containers outside the home<br>• Other: specify<br>• অন্যান্যঃ নির্দিষ্ট<br>• Does not know<br>• জানিনা<br>• Does not want to answer<br>• উত্তর প্রদানে অনিচ্ছুক
Section 4: Social capital (SC) – asked at baseline and follow-up<br>সেকশন ৪ঃ সামাজিক মূলধন এবং গবেষণা কার্যক্রমের গ্রহণযোগ্যতা (SC)- বেসলাইন এবং ফলো-আপে জিজ্ঞাসা করার জন্য		
SC1.	In the last 12 months, have you or adult members of your household been an active member (regularly do an activity or talk about things) of any of the following types of groups in your community?<br>গত ১২ মাসে, আপনি বা আপনার পরিবারের প্রাপ্তবয়স্ক সদস্যরা কি আপনার কমিউনিটির উল্লেখিত কোন ধরণের গ্রুপের সক্রিয়	• Community organization (e.g., residents' group)<br>• কমিউনিটি (এলাকা) ভিত্তিক প্রতিষ্ঠান (যেমন, বাসা বাড়ির গ্রুপ)<br>• Food distribution group<br>• খাবার বিতরণ গ্রুপ<br>• Political group<br>• রাজনৈতিক গ্রুপ

	সদস্য (নিয়মিত কোন কাজ করেন বা কোন বিষয় সম্পর্কে কথা বলেন) হিসেবে ছিলেন?	• Professional group or union<br>• পেশাদার গ্রুপ বা ইউনিয়ন<br>• Sports/social group<br>• ক্রীড়া/ সামাজিক গ্রুপ<br>• Religious group<br>• ধার্মিক গ্রুপ<br>• Women's group<br>• নারীদের গ্রুপ<br>• Committees for health, water, or electricity<br>• স্বাস্থ্য, পানি বা বিদ্যুতের জন্য কমিটি<br>• School groups<br>• স্কুল গ্রুপ<br>• Other: specify<br>• অন্যান্যঃ নির্দিষ্ট করে বলুন<br>• Does not know<br>• জানিনা<br>• Does not want to answer<br>• উত্তর প্রদানে অনিচ্ছুক<br>• Did not answer<br>• উত্তর দেননি
SC2.	In the last 12 months, did you or other adult members of your household receive from the group any emotional help, economic help or assistance in helping you know or do things?<br><br>গত ১২ মাসে, আপনি বা আপনার পরিবারের অন্যান্য প্রাপ্তবয়স্ক সদস্যরা কি উল্লেখিত গ্রুপগুলো থেকে কোনো মানসিক সাহায্য, অর্থনৈতিক সাহায্য পেয়েছেন বা তারা, কোন কিছুর ব্যাপারে জানতে বা কাজ সম্পন্ন করতে আপনাকে সাহায্য করেছে?<br>*Possible prompts: emotional help = emotional support; economical help = $, financial planning; in understanding something (e.g., how to breastfeed) or obtaining help (e.g., childcare support).*<br><br>বোঝানোর জন্য সম্ভাব্য ব্যাখ্যাগুলো:<br>মানসিক সহযোগিতা = মানসিক সমর্থন এর যোগান দেওয়া;<br>অর্থনৈতিক সাহায্য = অর্থ ধার বা আর্থিক পরিকল্পনা করতে সাহায্য;<br>কোন কিছুর ব্যাপারে জানতে সাহায্য করা (যেমন, কিভাবে স্তন্যপান করাবেন) অথবা সাহায্য পেতে সহায়তা করা (যেমন, চাইল্ড কেয়ার সাপোর্ট)।	• Community organization (e.g., residents' group)<br>• কমিউনিটি (এলাকা) ভিত্তিক প্রতিষ্ঠান (যেমন, বাসা বাড়ির গ্রুপ)<br>• Food distribution group<br>• খাবার বিতরণ গ্রুপ<br>• Political group<br>• রাজনৈতিক গ্রুপ<br>• Professional group or union<br>• পেশাদার গ্রুপ বা ইউনিয়ন<br>• Sports/social group<br>• ক্রীড়া/ সামাজিক গ্রুপ<br>• Religious group<br>• ধার্মিক গ্রুপ<br>• Women's group<br>• নারীদের গ্রুপ<br>• Committees for health, water, or electricity<br>• স্বাস্থ্য, পানি বা বিদ্যুতের জন্য কমিটি<br>• School groups<br>• স্কুল গ্রুপ<br>• Other: specify<br>• অন্যান্যঃ নির্দিষ্ট করে বলুন<br>• Does not know<br>• জানিনা

		• Does not want to answer<br>• উত্তর প্রদানে অনিচ্ছুক
SC3.	Of all these groups to which you or adult members of your household belong, which are the top three most important for the household?<br>এইসব গ্রুপ যাদের সাথে আপনি বা আপনার পরিবারের কোন সদস্য যুক্ত আছেন, তাদের মাঝে কোন ৩টি গ্রুপকে আপনার খানা/বাসার জন্য সবচেয়ে গুরুত্বপূর্ণ বলে মনে করেন?	• 1st: (name)<br>• প্রথমঃ (নাম)<br>• 2nd: (name)<br>• দ্বিতীয়ঃ (নাম)<br>• 3rd: (name)<br>• তৃতীয়ঃ (নাম)<br>Or<br>অথবা<br>• There are no important groups to this household<br>• কোন গুরুত্বপূর্ণ গ্রুপ নেই এই পরিবারের জন্য<br>• Does not know<br>• জানিনা<br>• Does not want to answer<br>• উত্তর প্রদানে অনিচ্ছুক
SC4.	In the last 12 months, have you received any help or support from any of the following, this can be emotional help, economic help or assistance in helping you know or do things?<br>গত ১২ মাসে, আপনি বা আপনার পরিবারের অন্যান্য প্রাপ্তবয়স্ক সদস্যরা কি উল্লেখিত গ্রুপগুলো থেকে কোনো মানসিক সাহায্য, অর্থনৈতিক সাহায্য পেয়েছেন বা কোন কাজ জানতে বা করতে আপনাকে সাহায্য করেছে?	• Family members outside the household<br>• বাসার বাহিরে পরিবারের সদস্যরা<br>• Neighbours<br>• প্রতিবেশী<br>• Friends who are not neighbours<br>• বন্ধু যারা প্রতিবেশী নন<br>• Community leaders<br>• কমিউনিটির নেতৃবৃন্দ<br>• Religious leaders<br>• ধর্মীয় নেতৃবৃন্দ<br>• Politicians<br>• রাজনীতিবিদ<br>• Government officials/civil service<br>• সরকারী অফিসার/ সিভিল সার্ভিস<br>• Charitable organisations/NGO<br>• দাতব্য প্রতিষ্ঠান/ এনজিও<br>• Other: specify<br>• অন্যান্যঃ নির্দিষ্ট করুন<br>• Does not know<br>• জানিনা<br>• Does not want to answer<br>• উত্তর প্রদানে অনিচ্ছুক
SC5.	In general, do you agree or disagree with the following statement:<br><br>Most people in this neighborhood are willing to help if you need it.	• Strongly agree<br>• দৃঢ়ভাবে একমত<br>• Agree somewhat<br>• কিছুটা একমত<br>• Neither agree or disagree<br>• সহমত বা দ্বিমত কিছুইনা

	সাধারণভাবে, আপনি কি নিম্নোক্ত মন্তব্যের সাথে সহমত বা দ্বিমত পোষণ করেনঃ<br>আপনার প্রয়োজনে অধিকাংশ প্রতিবেশী সাহায্য করতে ইচ্ছুক।<br><br>*Possible prompts: emotional help/support; economic help, lend money/financial planning; help in understanding something (e.g., how to breastfeed) or obtaining help (e.g., childcare support).*<br>বোঝানোর জন্য সম্ভাব্য ব্যাখ্যাগুলো:<br>মানসিক সহযোগিতা = মানসিক সমর্থন এর যোগান দেওয়া;<br>অর্থনৈতিক সাহায্য = অর্থ ধার বা আর্থিক পরিকল্পনা করতে সাহায্য;<br>কোন কিছুর ব্যাপারে জানতে সাহায্য করা (যেমন, কিভাবে স্তন্যপান করাবেন) অথবা সাহায্য পেতে সহায়তা করা (যেমন, চাইল্ড কেয়ার সাপোর্ট)।	• Disagree somewhat<br>• কিছুটা দ্বিমত<br>• Disagree strongly<br>• দৃঢ়ভাবে দ্বিমত<br>• Does not know<br>• জানিনা<br>• Does not want to answer<br>• উত্তর প্রদানে অনিচ্ছুক
SC6.	In general, do you agree or disagree with the following statement:<br><br>The majority of people in this community generally get along with each other.<br>সাধারণভাবে, আপনি কি নিম্নোক্ত মন্তব্যের সাথে সহমত বা দ্বিমত পোষণ করেনঃ<br><br>এই কমিউনিটির অধিকাংশ মানুষ সাধারণত একে অপরের সাথে মিলেমিশে থাকে	• Strongly agree<br>• দৃঢ়ভাবে একমত<br>• Agree somewhat<br>• কিছুটা একমত<br>• Neither agree or disagree<br>• সহমত বা দ্বিমত কিছুইনা<br>• Disagree somewhat<br>• কিছুটা দ্বিমত<br>• Disagree strongly<br>• দৃঢ়ভাবে দ্বিমত<br>• Does not know<br>• জানিনা<br>• Does not want to answer<br>• উত্তর প্রদানে অনিচ্ছুক
SC7.	In general, do you agree or disagree with the following statement:<br><br>The majority of people in this community would try and take advantage of you if they got the chance.<br>সাথে সহমত বা দ্বিমত পোষণ করেনঃ<br>এই কমিউনিটির অধিকাংশ মানুষ সুযোগ পেলে আপনার থেকে সুবিধা নেওয়ার চেষ্টা করবে।	• Strongly agree<br>• দৃঢ়ভাবে একমত<br>• Agree somewhat<br>• কিছুটা একমত<br>• Neither agree or disagree<br>• সহমত বা দ্বিমত কিছুইনা<br>• Disagree somewhat<br>• কিছুটা দ্বিমত<br>• Disagree strongly<br>• দৃঢ়ভাবে দ্বিমত<br>• Does not know<br>• জানিনা<br>• Does not want to answer<br>• উত্তর প্রদানে অনিচ্ছুক

SC8.	In general, do you agree or disagree with the following statement:<br><br>You feel that you are really part of the community.<br>সাধারণভাবে, আপনি কি নিম্নোক্ত মন্তব্যের সাথে সহমত বা দ্বিমত পোষণ করেনঃ<br><br>আপনি নিজেকে সত্যিকারর্থে কমিউনিটির অংশ মনে করেন	• Strongly agree<br>• দৃঢ়ভাবে একমত<br>• Agree somewhat<br>• কিছুটা একমত<br>• Neither agree or disagree<br>• সহমত বা দ্বিমত কিছুইনা<br>• Disagree somewhat<br>• কিছুটা দ্বিমত<br>• Disagree strongly<br>• দৃঢ়ভাবে দ্বিমত<br>• Does not know<br>• জানিনা<br>• Does not want to answer<br>• উত্তর প্রদানে অনিচ্ছুক
SC9.	In general, do you agree or disagree with the following statement:<br><br>You feel that you can trust Local/municipal government officials.<br>সাধারণভাবে, আপনি কি নিম্নোক্ত মন্তব্যের সাথে সহমত বা দ্বিমত পোষণ করেনঃ<br><br>আপনি মনে করেন যে, স্থানীয়/পৌরসভার সরকারি কর্মকর্তাদের বিশ্বাস করা যায়	• Strongly agree<br>• দৃঢ়ভাবে একমত<br>• Agree somewhat<br>• কিছুটা একমত<br>• Neither agree or disagree<br>• সহমত বা দ্বিমত কিছুইনা<br>• Disagree somewhat<br>• কিছুটা দ্বিমত<br>• Disagree strongly<br>• দৃঢ়ভাবে দ্বিমত<br>• Does not know<br>• জানিনা<br>• Does not want to answer<br>• উত্তর প্রদানে অনিচ্ছুক
SC10.	In the last 12 months, have you joined together with other community members to address a common problem or issue?<br>গত ১২ মাসে, আপনি কি এলাকার অন্যান্য মানুষদের সাথে মিলে কোন সমস্যার সমাধানের জন্য কাজ করেছেন?	• Yes<br>• হ্যাঁ<br>• No<br>• না<br>• Does not know<br>• জানিনা<br>• Does not want to answer<br>• উত্তর প্রদানে অনিচ্ছুক
SC11.	In the past 12 months, have you talked with a local authority or governmental organization about problems in this community?<br>গত ১২ মাসে, আপনি কি এই সম্প্রদায়ের সমস্যাগুলি সম্পর্কে স্থানীয় কর্তৃপক্ষ বা সরকারী সংস্থার সাথে কথা বলেছেন?	• Yes<br>• হ্যাঁ<br>• No<br>• না<br>• Does not know<br>• জানিনা<br>• Does not want to answer<br>• উত্তর প্রদানে অনিচ্ছুক
SC12.	In general, how do you feel about the security in your neighborhood?	• Very safe<br>• খুবি নিরাপদ

	সাধারণভাবে, আপনার চারপাশের এলাকায় আপনি কতটুকু নিরাপদ অনুভব করেন?	• Moderately safe<br>• মোটামুটি নিরাপদ<br>• Neither safe or unsafe<br>• নিরাপদ বা অনিরাপদ কোনটিই নয়<br>• Moderately unsafe<br>• মোটামুটি অনিরাপদ<br>• Very unsafe<br>• খুবি অনিরাপদ<br>• Does not know<br>• জানিনা<br>• Does not want to answer<br>• উত্তর প্রদানে অনিচ্ছুক
SC13.	Compared to someone at your age and sex, how do you consider your health?<br>আপনার সমবয়সী এবং একই লিঙ্গের অন্য কারো তুলনায় আপনি নিজের স্বাস্থ্যকে কিভাবে বিবেচনা করেন?	• Very good<br>• খুবি ভাল<br>• Good<br>• ভাল<br>• Fair<br>• ফেয়ার/ যথাযথ<br>• Bad<br>• খারাপ<br>• Very bad<br>• খুব খারাপ<br>• Does not know<br>• জানিনা<br>• Does not want to answer<br>• উত্তর প্রদানে অনিচ্ছুক
Section 5: Dengue knowledge, attitudes, and practices (KAP) – asked at baseline and follow-up<br>সেকশন ৫ঃ ডেঙ্গু সম্পর্কিত জ্ঞান, মনোভাব, এবং অভ্যাস - বেসলাইন ও ফলো-আপ এ প্রশ্ন করা হবে		
KAP1.	Have you heard of dengue?<br>আপনি কি ডেঙ্গুর ব্যাপারে শুনেছেন?	• Yes (হ্যাঁ)<br>• No (skip to KAP5) (না, KAP5 এ চলে যান)
KAP2.	Do you know what dengue is?<br>আপনি কি জানেন ডেঙ্গু কি?<br>*Do not read the options. Select all that apply.*<br>*উত্তরগুলো পড়ে শোনাবেন না/ সবগুলো উত্তর চিহ্নিত করুন*	• Dengue is a viral disease (ডেঙ্গু একটি ভাইরাসজনিত রোগ)<br>• Dengue is spread by mosquitoes (ডেঙ্গু মশার মাধ্যমে ছড়ায়)<br>• Aedes is the mosquito responsible for spreading Dengue (এডিস মশার মাধ্যমে ডেঙ্গু ছড়ায়)<br>• Other (অন্যান্য)
KAP3.	What do you think are the main signs and symptoms of dengue?<br>আপনার মতে ডেঙ্গু রোগের প্রধান লক্ষণ ও উপসর্গ গুলো কি?<br>*Do not read the options.*<br>*Alternate phrasing: If you or a family member have dengue, how do you know? Do you see certain signs of sickness?*<br>*উত্তরগুলো পড়ে শোনাবেন না/*	• Fever (জ্বর)<br>• Headache (মাথা ব্যাথা)<br>• Bone pain (হার ব্যাথা)<br>• Sore muscles (পেশীতে ব্যাথা)<br>• Rash (red spots on skin) (ফুসকুড়ি বা চামড়ায় লাল দাগ দেখা)<br>• Stomach pain (পেটে ব্যাথা)

	অন্যভাবে বলার জন্যঃ<br>*যদি আপনার বা আপনার পরিবারের কারো ডেঙ্গু হয়ে থাকে তাহলে আপনি কিভাবে সেটা বুঝতে পারেন? আপনি কি কোন বিশেষ লক্ষণ দেখতে পারেন সেক্ষেত্রে?*	● Eye pain (চোখে ব্যাথা)<br>● Nausea and/or vomiting (বমি বমি ভাব এবং/অথবা বমি হওয়া)<br>● Sweating (ঘাম হওয়া)<br>● Bleeding gums (মাড়িতে রক্তপাত)<br>● Nosebleeds (নাক দিয়ে রক্ত পড়া)<br>● Diarrhea (ডায়রিয়া)<br>● Coughing (কাশি)<br>● Weakness (দূর্বলতা)<br>● Other: specify (অন্যান্যঃ নির্দিষ্ট করুন)<br>● Does not know (জানিনা)<br>● Does not want to answer (উত্তর দিতে চাই না)<br>● Did not answer (উত্তর দেয়নি)
KAP4.	In your opinion, how is dengue spread?<br>*Do not read the options*<br>আপনার মতে ডেঙ্গু কিভাবে ছড়ায়?<br>*উত্তরগুলো পড়ে শোনাবেন না*	● By mosquitoes (মশার মাধ্যমে)<br>● By contact with infected people (আক্রান্ত ব্যাক্তির সংস্পর্শে আসলে)<br>● By unhygienic food (অস্বাস্থ্যকর খাবার খেলে)<br>● By unboiled water (না ফুটানো জল/পানি খেলে)<br>● By bad weather (খারাপ আবহাওয়া থেকে)<br>● By visiting rural/farming areas where there are cows and pigs (গ্রামীণ/খামার এলাকা পরিদর্শন করে যেখানে গরু এবং শূকর আছে)<br>● By skin contact (ত্বকের স্পর্শের মাধ্যমে)<br>● By intimate contact (অন্তরঙ্গ যোগাযোগের মাধ্যমে)<br>● By the air (বাতাসের মাধ্যমে)<br>● By water (পানি/জলের মাধ্যমে)<br>● Other: specify (অন্যান্যঃ নির্দিষ্ট করুন)<br>● Does not know (জানিনা)<br>● Does not want to answer (উত্তর দেয়নি)
KAP5.	Dengue is a disease spread by mosquitoes. Where in your community do you often see mosquitoes ?<br>ডেঙ্গু মশা দ্বারা ছড়ানো একটি রোগ। আপনি আপনার এই এলাকায় প্রধান মশার প্রজনন বা ডিম পাড়ার স্থান কোথায় বলে মনে করেন?<br>*Do not read the options.*<br>*উত্তরগুলো পড়ে শোনাবেন না*	● Water containers in households (বাড়িঘরের পানি জমা রাখার পাত্র)<br>● Trash in the community (এলাকার ময়লা-আবর্জনা থেকে)<br>● Sewers in the community (এলাকার পয়ঃনিষ্কাশনের ক্ষেত্র থেকে)<br>● Vacant lots (শূন্য এলাকা থেকে)<br>● Abandoned houses (পরিত্যাক্ত বাসা)<br>● Garbage dumps in the street (রাস্তায় ময়লা ফেলার স্থান)<br>● Domestic organic garbage dumps (গৃহস্থালির জৈব আবর্জনা ফেলার স্থান)

		● Tires (dumped in the neighbourhood) (গাড়ির টায়ার, যেটি পার্শবর্তী এলাকায় ফেলে দেওয়া হয়েছে)<br>● Car repair shops (গাড়ি মেরামতের কারখানা)<br>● Construction sites (নির্মান এলাকা)<br>● Scrap yards (ভাঙ্গারির এলাকা)<br>● Other: specify (অন্যান্যঃ নির্দিষ্ট করুন)<br>● Does not know (জানিনা)<br>● Does not want to answer (উত্তর দেয়নি)
KAP6.	In your opinion, are there ways that dengue can be prevented?<br>আপনার মতে ডেঙ্গু কে কি প্রতিরোধ করা সম্ভব?<br>*Alternate phrasing: do you think you or other people can prevent dengue?*<br>*অন্যভাবে বললেঃ আপনি কি মনে করেন যে আপনি বা অন্যরা কোনভাবে ডেঙ্গুকে প্রতিরোধ করতে পারবেন?*	● Yes (হ্যাঁ)<br>● No (skip to KAP8) (না- KAP8 এ যান)<br>● Does not know (skip to KAP8) (জানিনা – KAP8 এ যান)<br>● Does not want to answer (skip to KAP8) (উত্তর দিতে চান না – KAP8 এ যান)
KAP7.	Could you tell me what people should do to prevent dengue?<br>আপনি কি বলতে পারেন যে ডেঙ্গু কে প্রতিরোধ করতে হলে কি করতে হবে?<br><br>*Do not read the options.*<br>*উত্তর পড়ে শোনাবেন না।*<br>*Alternate phrasing: what can be done to reduce the amount of mosquitoes in your home or in the community?*<br>*অন্যভাবে বললেঃ কিভাবে আপনার বাসায় বা এলাকায় মশার সংখ্যা কমানো যেতে পারে?*	● Keep water in closed water storage containers (ঢাকনাসহ পানি রাখার পাত্রে পানি রাখা হয়েছে)<br>● Use larvicide in containers (বাসার পানি রাখার পাত্রে লার্ভিসাইড ব্যবহার করা হয়েছে- সেক্ষেত্রে লার্ভিসাইডের নাম উল্লেখ করুন)<br>● Fumigate inside or outside of home (বাসার ভেতরে ধোয়া দেওয়া হয়েছে)<br>● Clean up breeding grounds (e.g. clean up debris and trash, clean containers, emptying water from containers) (বাসার ভেতরে মশার বংবৃদ্ধির স্থান পরিষ্কার করা হয়েছে)<br>● Other: specify<br>● Does not know (জানিনা)<br>● Does not want to answer (উত্তর দেয়নি)
KAP8.	If you or someone in your family suspects dengue, what do you do?<br>যদি আপনার বা আপনার পরিবারের কারো ডেঙ্গু হয়েছে বলে ধারনা করেন, তখন আপনি কি করেন?<br>*Do not read the options.*<br>*উত্তরগুলো পড়ে শোনাবেন না*<br>*Alternate phrasing/prompting: Do you take anything or get help? If so, what and where?*<br>*আপনি কি কোথাও থেকে সাহায্য চান বা কিছু গ্রহণ করেন প্রতিষেধক হিসেবে? হ্যাঁ হলে কি এবং কোথায়?*	● Do nothing (কিছুই করি না)<br>● Take headache/fever medicine (মাথাব্যাথা/জ্বরের জন্য ওষুধ নেই)<br>● Increase liquid intake (keep hydrated) (হাইড্রেট থাকার জন্য তরল খাবার গ্রহণের পরিমাণ বাড়িয়ে দেই)<br>● Go to the hospital (হাসপাতালে চলে যাই)<br>● Go to the clinic or see a doctor (ক্লিনিকে বা ডাক্তারের কাছে দেখাতে যাই)<br>● Go to the pharmacy (ফার্মাসি তে যাই)<br>● Other: specify (অন্যান্যঃ নির্দিষ্ট করুন)<br>● Does not know (জানিনা)<br>● Does not want to answer (উত্তর দেয়নি)

Supplement 1
Questionnaire, KAP score. Household index and Social Capital index

KAP9.	In your opinion, do you think that dengue is a problem for you and your household?<br>আপনার মতে ডেঙ্গু কি আপনার বা আপনার পরিবারের জন্য একটি হুমকি?	● Yes strongly agree (হ্যাঁ, দৃঢ়ভাবে একমত)<br>● Yes somewhat agree (হ্যাঁ, কিছুটা একমত)<br>● Neither yes or no (সহমত বা দ্বিমত কিছুইনা)<br>● No somewhat disagree (না, কিছুটা দ্বিমত)<br>● No strongly disagree (না, দৃঢ়ভাবে দ্বিমত)<br>● Does not know (জানিনা)<br>● Does not want to answer (উত্তর প্রদানে অনিচ্ছুক)
KAP10.	What are your and your family's main sources of information about mosquito control and dengue?<br>ডেঙ্গু এবং মশার বৃদ্ধি রোধের ব্যাপারে আপনার ও আপনার পরিবারের তথ্য প্রাপ্তির প্রধান উৎস কি?	● Family members or friends (পরিবারের মানুষজন)<br>● Doctor, nurse (ডাক্তার, সেবিকা)<br>● School (স্কুল)<br>● Entomological/dengue agent (কীটতত্ত্ববিদ/ডেঙ্গু বিষয়ক তথ্যদাতা)<br>● Health agent (স্বাস্থ্যকর্মী)<br>● Other professional: specify (অন্যান্য কর্মীঃ নির্দিষ্ট করুন)<br>● Community leader (এলাকার নেতা)<br>● Religious leader (ধর্মীয় নেতা)<br>● Television (টেলিভিশন)<br>● Newspapers (দৈনিক পত্রিকা)<br>● Radio (রেডিও)<br>● Social media and messaging (WhatsApp, telegram, Messenger)<br>● Internet searches/browsing (ইন্টারনেট)<br>● Other: specify (অন্যান্যঃ নির্দিষ্ট করুন)<br>● Does not know (জানিনা)<br>● Does not want to answer (উত্তর দেয়নি)
KAP11.	How much danger do you feel that you have of getting sick from dengue fever?<br>ডেঙ্গু জ্বরে আক্রান্ত হবার জন্য আপনি কতটুকু ঝুঁকিতে আছেন বলে আপনি মনে করেন?	● All of the time (সবসময়)<br>● Most of the time (বেশিরভাগ সময়)<br>● Some of the time (মাঝে মাঝে)<br>● A little of the time (খুব অল্প সময়ে)<br>● None of the time (কখনো না)<br>● Does not know (জানিনা)<br>● Does not want to answer (উত্তর দেয়নি)
KAP12.	In this house, in the last 30 days, what did you/your household do to reduce the presence of mosquitos?<br>*এই বাড়িতে গত ৩০ দিনে মশার সংখ্যা কমানোর জন্য কি কি পদেক্ষেপ নেওয়া হয়েছে?*<br>*Do not read the options.*<br>*উত্তরগুলো পড়ে শোনাবেন না*	● Did nothing (কিছুই করা হয় নি)<br>● Kept water in closed water storage containers (ঢাকনাসহ পানি রাখার পাত্রে পানি রাখা হয়েছে)<br>● Used larvicide in containers (বাসার পানি রাখার পাত্রে লার্ভিসাইড ব্যবহার করা হয়েছে- সেক্ষেত্রে লার্ভিসাইডের নাম উল্লেখ করুন)<br>● Fumigated inside or outside of home (বাসার ভেতরে ধোয়া দেওয়া হয়েছে)

		• Cleaned up breeding grounds (e.g. clean up debris and trash, clean containers, emptying water from containers) (বাসার ভেতরে মশার বংবৃদ্ধির স্থান পরিষ্কার করা হয়েছে)<br>• Other: specify<br>• Does not know (জানিনা)<br>• Does not want to answer (উত্তর দেয়নি)
KAP13.	Do you think that your community can prevent dengue on its own?<br>আপনার কি মনে হয় যে আপনার এলাকার মানুষজন নিজেদের থেকেই ডেঙ্গু প্রতিরোধে সক্ষম?	• Yes strongly agree (হ্যাঁ, দৃঢ়ভাবে একমত)<br>• Yes somewhat agree (হ্যাঁ, কিছুটা একমত)<br>• Neither yes or no (সহমত বা দ্বিমত কিছুইনা)<br>• No somewhat disagree (না, কিছুটা দ্বিমত)<br>• No strongly disagree (না, দৃঢ়ভাবে দ্বিমত)<br>• Does not know (জানিনা)<br>• Does not want to answer (উত্তর প্রদানে অনিচ্ছুক)
KAP14.	Since May 2023, have workers from the municipality taken action to control mosquitoes in your home and/or community?<br>গত মে ২০২২ থেকে পৌরসভার থেকে কি আপনার বাসায়/এলাকায় মশা দমন বা প্রতিরোধের জন্য কোন ব্যবস্থা নেওয়া হয়েছে?	• Yes (হ্যাঁ)<br>• No<br>• Does not know (জানিনা)
KAP15.	If mosquito control workers from the municipality came to your house, would you allow them to enter and look for mosquitoes?<br>যদি পৌরসভা থেকে মশা রোধের জন্য কর্মীরা আপনার বাসায় আসে, আপনি কি তাদের ভেতরে প্রবেশ করে মশা খোজার জন্য অনুমতি দেবেন?	• Yes (হ্যাঁ)<br>• No (না)<br>• Does not know (জানিনা)<br>• Does not want to answer (উত্তর প্রদানে অনিচ্ছুক)
KAP16.	Where did mosquito control workers from the municipality take action to control mosquitoes?<br>পৌরসভার কর্মিরা মশারোধের জন্য কোথায় কোথায় ব্যবস্থা নিয়েছে?	• In my home and community (আমার বাসায় ও এলাকায়)<br>• In my home only (শুধু আমার বাসায়)<br>• In my community only (শুধু আমার এলাকায়)<br>• Does not know (জানিনা)
KAP17.	What actions did they take to control mosquitoes?<br>তারা কি ধরনের ব্যবস্থা নিয়েছে?	• Fumigated inside home (বাসার ভেতরে ধোয়া দেওয়া)<br>• Fumigated outside home/on the street where you live (যেখানে আমি বাস করি সেই বাসার বাইরে/রাস্তায় ধোয়া দেওয়া)
KAP18.	Do you think the actions of the mosquito control workers from the municipality helped to control mosquitoes?	• Yes strongly agree (হ্যাঁ, দৃঢ়ভাবে একমত)

Supplement 1
Questionnaire, KAP score. Household index and Social Capital index

Cross-sectional study on the association between seroprevalence among children and their parents' knowledge, attitudes, and practices in a dengue-endemic city in Bangladesh

	আপনার কি মনে হয় যে মশার বিস্তার রোধে পৌরসভার কর্মীদের নেওয়া পদক্ষেপ কার্যকর হয়েছে?	● Yes somewhat agree (হ্যাঁ, কিছুটা একমত)<br>● Neither yes or no (সহমত বা দ্বিমত কিছুইনা)<br>● No somewhat disagree (না, কিছুটা দ্বিমত)<br>● No strongly disagree (না, দৃঢ়ভাবে দ্বিমত)<br>● Does not know (জানিনা)<br>● Does not want to answer (উত্তর প্রদানে অনিচ্ছুক)
Section 6: Dengue episodes (DEN) – asked at baseline and follow-up<br>সেকশন ৬ঃ ডেঙ্গু ঘটার এপিসোডগুলো (DEN) - বেসলাইন ও ফলো-আপ এ প্রশ্ন করতে হবে		
DEN1.	How many people have suffered from a dengue fever episode since the last dengue season (May 2023) in this household (not including participating children)?<br>আপনার বাসায় গত ডেঙ্গু সিজন থেকে (মে ২০২২) কতজন ডেঙ্গু রোগে ভুগেছেন (অংশগ্রহণকারী বাচ্চাদের বাদ দিয়ে)<br><br>*DEN2 through DEN9 asked for each person who suffered from a dengue fever episode.*<br>*DEN2 থেকে DEN9 তাদের সবাইকে প্রশ্ন করতে হবে যাদের ডেঙ্গু হয়েছিলো*	● ____ people (if 0, skip to DEN10) (__________ জন মানুষ, যদি ০ হয় তাহলে DEN10 এ যান)<br>● Does not know (skip to DEN10) (জানিনা, DEN10 এ যান)<br>● Does not want to answer (skip to DEN10) (উত্তর দিতে চান নি, DEN10 এ যান)
DEN2.	*if answered any number in DEN1;*<br>যদি *DEN1 এ কোন নাম্বার উল্লেখ করে থাকেন;*<br>What is the age of this person (who has had a dengue fever episode since May 2023)?<br>ঐ মানুষটির বয়স কতো (যার গত মে ২০২২ এর পড় থেকে কখনো ডেঙ্গু হয়েছিলো)?	● ___ years (_______ বছর)<br>● Does not know (জানিনা)<br>● Refused (বলতে চায় নি)
DEN3.	What were the symptoms the person experienced during the dengue episode?<br>ডেঙ্গু রোগে আক্রান্ত থাকার সময় ওনার কি লক্ষণ ছিলো?<br>[answer Yes/No/Does not know to all]<br>(হ্যা/না/জানিনা সবার জন্য উত্তর যোগ করুন)<br><br>[If they don't mention bleeding, vomiting, bloody feces/urine, ask if any of these were present]	● Rash (ফুসকুড়ি বা চামড়ায় লাল দাগ দেখা)<br>● Bruising (ক্ষত)<br>● Fatigue/weakness (দূর্বলতা)<br>● Headaches or neck pain (ঘাড় ও মাথা ব্যাথা)<br>● Pain in or behind the eyes (চোখে বা চোখের পেছনে ব্যাথা)<br>● Joint pain (শরীরের সংযোগে ব্যথা)<br>● Muscle pain (পেশীতে ব্যাথা)<br>● Dry/oily couh (শুষ্ক/তৈলাক্ত কাশি)<br>● Difficulty breathing (নিঃশ্বাস নিতে সমস্যা)<br>● Nasal congestion (বন্ধ নাক)<br>● Sore throat (গলা ব্যাথা)<br>● Bleeding nose or gums (নাক ও মাড়িতে রক্তপাত)

		• Nausea/vomiting (বমি বমি ভাব এবং/অথবা বমি হওয়া)<br>• Bloody vomiting (রক্ত বমি হওয়া)<br>• Loss of appetite (ক্ষুধামন্দা)<br>• Abdominal pain (পেটে ব্যাথা)<br>• Diarrhea (ডায়রিয়া)<br>• Constipation (কোষ্ঠকাঠিন্য)<br>• Bloody feces/urine (রক্তাক্ত মল/মূত্র)
DEN4.	Was hospitalization required for the dengue fever episode since May 2023?<br>মে ২০২২ সাল থেকে কি ডেঙ্গু জ্বর পর্বের জন্য কোন হাসপাতালে ভর্তির প্রয়োজন ছিল?<br>*Hospitalization meaning staying overnight.*<br>*হাসপাতালে ভর্তি মানে রাতের বেলা থাকা*	• Yes (হ্যাঁ)<br>• No (না)<br>• Does not know (জানিনা)<br>• Does not want to answer (উত্তর দিতে চায় নি)
DEN5.	Was hemorrhagic dengue fever diagnosed?<br>ঐ সময় কি হেমোরেজিক ডেঙ্গু জ্বর নির্ণয় করা হয়েছিল?	• Yes (হ্যাঁ)<br>• No (না)<br>• Does not know (জানিনা)<br>• Does not want to answer (উত্তর দিতে চায় নি)
DEN6.	Does this person have any known chronic disease or other health condition?<br>এই মানুষটির কি অন্য কোন দীর্ঘস্থায়ী রোগ বা অন্য স্বাস্থ্যগত সমস্যা ছিলো আগের থেকে?	• Yes (হ্যাঁ)<br>• No (skip to DEN8) (না, তাহলে DEN8 এ যান)<br>• Does not know (skip to DEN8) (জানিনা, তাহলে DEN8 এ যান)<br>• Does not want to answer (skip to DEN8) (উত্তর দিতে চান না, তাহলে DEN8 এ যান)
DEN7.	If yes, please specify<br>হ্যাঁ হলে নির্দিষ্ট করুন,	• Diabetes (ডায়াবেটিস/বহুমূত্র)<br>• Cancer (ক্যান্সার)<br>• Heart disease (হৃদরোগ)<br>• Lupus (লুপাস)<br>• Rheumatoid arthritis (রিউমাটয়েড আর্থ্রাইটিস)<br>• Multiple sclerosis (মাল্টিপল স্ক্লেরোসিস)<br>• Other: specify (অন্যান্যঃ নির্দিষ্ট করুন)<br>• Does not know (জানিনা)<br>• Does not want to answer (উত্তর দেয়নি)
DEN8.	Can you recall if anyone in your family ever suffered from dengue fever in their lifetime? Your family includes not only the people that live with you, but any siblings or other family members.<br>[skipped if DEN1 > 0]<br>আপনি কি মনে করতে পারেন আপনার পরিবারের কেউ তাদের জীবদ্দশায় ডেঙ্গু জ্বরে আক্রান্ত হয়েছে কিনা? (কেউ না হলে বা ০ হলে পরের সেকশনে চলে যান)	• Yes, someone suffered from dengue fever (হ্যাঁ, ডেঙ্গু জ্বরে আক্রান্ত হয়েছিলো)<br>• No, no one suffered from dengue fever (skip to SER1) (না, কেউ ডেঙ্গু জ্বরে আক্রান্ত হয় নি, তাহলে SER1 এ যান)<br>• Does not know (skip to SER1) (জানিনা, SER1 এ যান)<br>• Does not want to answer (skip to SER1) (উত্তর দিতে চান না, SER1 এ যান)

Supplement 1
Questionnaire, KAP score. Household index and Social Capital index

DEN9.	Can you recall if anyone in your family ever died from dengue fever? Your family includes not only the people that live with you, but any siblings or other family members.<br>[asked of all respondents]<br>আপনার মনে আছে কি আপনার পরিবারের কেউ ডেঙ্গু জ্বরে মারা গেছে?	● Yes, someone died from dengue fever (হ্যাঁ, ডেঙ্গু জ্বরে মারা গিয়েছিলো)<br>● No, no one died from dengue fever (skip to SER1) (না, কেউ ডেঙ্গু জ্বরে মারা যায় নি, তাহলে SER1 এ যান)<br>● Does not know (skip to SER1) (জানিনা, SER1 এ যান)<br>● Does not want to answer (skip to SER1) (উত্তর দিতে চান না, SER1 এ যান)
Section 7: Child Serology (SER)<br>সেশন ৭ঃ চাইল্ড সেরোলজি (SER)		
SER1.	How many children will participate with blood samples?<br><br>*Repeat SER2 through SER16 for the number of children indicated in SER1.*<br>*কতজন শিশু রক্তের নমুনা সংগ্রহে অংশগ্রহণ করবে?*<br><br>*SER1 এ নির্দেশিত শিশুদের সংখ্যার জন্য SER2 থেকে SER20 পুনরাবৃত্তি করুন।*	__ child(ren)<br>--- শিশু
SER2.	When is the child's birthdate?<br>শিশুটির জন্মদিন কখন?	_ _ / _ _ / _ _ (dd/mm/yy)<br>_ _ / _ _ / _ _ (দিন/মাস/বছর)
SER3.	What is the child's gender?<br>শিশুর লিঙ্গ কি?	● Male<br>● পুরুষ<br>● Female<br>● মহিলা<br>● Third gender<br>● হিজড়া<br>● Other<br>● অন্যান্য<br>● Does not want to answer<br>● উত্তর প্রদানে অনিচ্ছুক
SER4.	Does the child attend school or daycare outside the home?<br>শিশুটি কি স্কুল যায়?	● Yes<br>● হ্যাঁ<br>● No (skip to SER10)<br>● না (SER10 এ যান)<br>● Does not know (skip to SER10)<br>● জানিনা (SER10 এ যান)<br>● Does not want to answer (skip to SER10)<br>● উত্তর প্রদানে অনিচ্ছুক (SER10 এ যান)

Supplement 1
Questionnaire, KAP score. Household index and Social Capital index

Cross-sectional study on the association between seroprevalence among children and their parents' knowledge, attitudes, and practices in a dengue-endemic city in Bangladesh

SER5.	Does this child have any known chronic disease or other health condition?<br>এই শিশুর কি কোনো দীর্ঘস্থায়ী রোগ বা অন্যান্য স্বাস্থ্যগত সমস্যা আছে?	● Yes (হ্যাঁ)<br>● No (skip to SER7) (না, SER7 এ যান)<br>● Does not know (skip to SER7) (জানিনা, SER7 এ যান)<br>● Does not want to answer (skip to SER7) (উত্তর দিতে চান নি, SER7 এ যান)
SER6.	If yes, please specify:<br>যদি হ্যাঁ হয়, নির্দিষ্ট করুন	● Diabetes<br>● ডায়াবেটিস<br>● Cancer<br>● ক্যানসার<br>● Heart disease<br>● হৃদযন্ত্রের রোগ<br>● Lupus<br>● লুপুস<br>● Rheumatoid arthritis<br>● রিউম্যাটয়েড আর্থ্রাইটিস/ বাত<br>● Multiple sclerosis<br>● মাল্টিপল স্ক্লেরোসি<br>● Other: specify<br>● অন্যান্যঃ নির্দিষ্ট করুন<br>● Does not know<br>● জানিনা<br>● Does not want to answer<br>● উত্তর প্রদানে অনিচ্ছুক
SER7.	Since May 2023, has this child suffered from a dengue fever episode?<br>মে ২০২২ থেকে শিশুটি কি কোন সময় ডেঙ্গু জ্বরে ভুগেছে?	● Yes (হ্যাঁ)<br>● No (skip to next child/end) (না, তাহলে পরের বাচাটির ব্যাপারে জিজ্ঞেস করুন বা শেষ করুন)<br>● Does not know (skip to next child/end) (না, তাহলে পরের বাচাটির ব্যাপারে জিজ্ঞেস করুন বা শেষ করুন)<br>● Does not want to answer (skip to next child/end) (না, তাহলে পরের বাচাটির ব্যাপারে জিজ্ঞেস করুন বা শেষ করুন)
SER8.	Did the child experience the following symptoms during the dengue episode?<br><br>শিশুটি কি ডেঙ্গুর সময় উলেখিত উপসর্গে ভুগেছে?<br><br>[answer Yes/No/Does not know to all]<br>(উত্তর হ্যাঁ, না, একদম জানিনা)<br><br>[If they don’t mention bleeding, vomiting, bloody feces/urine, ask if any of these were present]	● Rash<br>● ফুসকুড়ি<br>● Bruising<br>● ক্ষত<br>● Fatigue/weakness<br>● ক্লান্তি/ দুর্বলতা<br>● Headaches or neck pain<br>● মাথাব্যথা বা ঘাড় ব্যথা<br>● Pain in or behind the eyes<br>● চোখের ভিতরে বা বাহিরে ব্যথা<br>● Joint pain<br>● জোড়ায় ব্যথা<br>● Muscle pain

		• পেশীর ব্যথা<br>• Dry/oily cough<br>• শুকনো/ভেজা কাশি<br>• Difficulty breathing<br>• শ্বাসকষ্ট<br>• Nasal congestion<br>• নাক বন্ধ<br>• Sore throat<br>• গলা ব্যথা<br>• Bleeding nose or gums<br>• নাক বা মাড়ি থেকে রক্ত বের হওয়া<br>• Nausea/vomiting<br>• বমি বমি ভাব/বমি করা<br>• Bloody vomiting<br>• রক্ত বমি করা<br>• Loss of appetite<br>• ক্ষুধা কমে যাওয়া<br>• Abdominal pain<br>• পেট ব্যথা<br>• Diarrhea<br>• ডায়রিয়া<br>• Constipation<br>• কোষ্ঠকাঠিন্য<br>• Bloody feces/urine<br>• রক্তাক্ত মল/ প্রস্রাব
SER9.	For the dengue episode, was medical attention sought ?<br>ডেঙ্গুর সময় উল্লেখিত কোন প্রতিষ্ঠান থেকে কি কোন চিকিৎসা সেবা নেওয়া হয়েছিল?	• Yes<br>• হ্যাঁ<br>• No<br>• না<br>• Does not know<br>• জানিনা<br>• Does not want to answer<br>• উত্তর প্রদানে অনিচ্ছুক
SER10.	Was hospitalization required for the dengue fever episode since May 2023?<br><br>মে ২০২২ থেকে ডেঙ্গুর সময় কি হাসপাতালে ভর্তি হতে হয়েছিল?<br><br>*Hospitalization meaning staying overnight.*<br>হাসপাতালে ভর্তি হওয়া বলতে রাতে অবস্থান করা	• Yes<br>• হ্যাঁ<br>• No<br>• না<br>• Does not know<br>• জানিনা<br>• Does not want to answer<br>• উত্তর প্রদানে অনিচ্ছুক
SER11.	Was hemorrhagic dengue fever diagnosed?<br>হেমোরেজিক ডেঙ্গু জ্বর কি হয়েছিল?	• Yes<br>• হ্যাঁ<br>• No<br>• না

		• Does not know<br>• জানিনা<br>• Does not want to answer<br>• উত্তর প্রদানে অনিচ্ছুক

NOTE: KAP16, KAP17 and KAP18 were not considered for the score in any step.

Supplement 1
Questionnaire, KAP score. Household index and Social Capital index

Cross-sectional study on the association between seroprevalence among children and their parents' knowledge, attitudes, and practices in a dengue-endemic city in Bangladesh

1.2 KAP score

Knowledge				
**#**	**Question**	**Answers options**	**Scoring method**	**score**
KAP1	Have you heard of dengue?	1= Yes<br>0= No	0= Incorrect<br>1= correct	1
KAP2	Do you know what dengue is?	1= Dengue is a viral disease<br>2= Dengue is spread by mosquitoes<br>3= Aedes is the mosquito responsible for spreading Dengue<br>0=I don't know much about it	2 if 2+ correct answers given; 1 if 1 correct answer<br>given; 0 otherwise.	2
KAP3	What do you think are the main signs and symptoms of dengue?	Fever<br>Headache<br>Bone pain<br>Sore muscles<br>Rash (red spots on skin)<br>Stomach pain<br>Eye pain<br>Nausea and/or vomiting<br>Sweating<br>Bleeding gums<br>Nosebleeds<br>Diarrhea<br>Coughing<br>Weakness<br>Does not know<br>Does not want to answer	3 if fever, nausea, aches/pains, and rash given;<br>2 if fever plus at least one of nausea, aches/pains, and<br>rash given;<br>1 if fever given but not nausea, aches/pains, and rash; 0 otherwise	3

KAP4	In your opinion, how is dengue spread?	By mosquitoes<br>By contact with infected people<br>By unhygienic food<br>By unboiled water<br>By bad weather<br>By visiting rural/farming areas where there are cows and pigs<br>By skin contact<br>By intimate contact<br>By the air<br>By water<br>Other: specify<br>Does not know<br>Does not want to answer	1 if it is by mosquitoes;<br>-0.5 if the answer is: by contact with infected people, or by unhygienic food, or by unboiled water, or by bad weather, or by visiting rural/farming areas where there are cows and pigs, or by skin contact, or by intimate contact, or by the air, or by water<br>0 if the answer is: does not know	1
KAP5	Dengue is a disease spread by mosquitoes. Where in your community do you often see mosquitoes ?	Water containers in households<br>Trash in the community<br>Sewers in the community<br>Vacant lots<br>Abandoned houses<br>Garbage dumps in the street<br>Domestic organic garbage dumps<br>Tires (dumped in the neighbourhood)<br>Car repair shops<br>Construction sites<br>Scrap yards<br>Other: specify<br>Does not know<br>Does not want to answer	1 if the answer is: water containers in households, or tires, or car repair shops, or construction sites, or scrap yards.<br>0 otherwise	1
KAP6	In your opinion, are there ways that dengue can be prevented?	1= Yes<br>0= No<br>0 = Does not know<br>0= Does not want to answer	1 if yes; 0 otherwise	1

KAP7	Could you tell me what people should do to prevent dengue?	Keep water in closed-water storage containers<br>Use larvicide in containers<br>Fumigate inside or outside of the home<br>Clean up breeding grounds (e.g. clean up debris and trash, clean containers, emptying water from containers)<br>Other:<br>0 = Does not know<br>0 = Does not want to answer	Sum of the following, divided by 3:<br>3 if the answer is: Keep water in closed-water storage containers, or use larvicide in containers, or fumigate inside or outside of the home, or clean up breeding grounds.<br>0 otherwise.	3
KAP10	What are your and your family's main sources of information about mosquito control and dengue?	Family members or friends<br>Doctor, nurse<br>School<br>Entomological/dengue agent<br>Health agent<br>Other professional: specify<br>Community leader<br>Religious leader<br>Television<br>Newspapers<br>Radio<br>Social media and messaging (WhatsApp, telegram, Messenger)<br>Internet searches/browsing<br>Other: specify<br>Does not know<br>Does not want to answer	2 points max*<br>1 for each following answers:<br>Doctor(nurse), school,<br>Entomological/dengue agent, Health agent, Health<br>center<br>0.5 for each following answers:<br>community leader,<br>friends, family, newspapers, television, radio,<br>Internet<br>0 otherwise(refused, does not know, religious<br>leader)<br><br>*if the sum is higher or equal to 2, the score is 2, otherwise, the score stays the same.	2

Supplement 1
Questionnaire, KAP score. Household index and Social Capital index

Cross-sectional study on the association between seroprevalence among children and their parents' knowledge, attitudes, and practices in a dengue-endemic city in Bangladesh

Attitudes				
#	Question	Answers	Scoring method	Score
KAP9	In your opinion, do you think that dengue is a problem for you and your household?	6= Strongly agree<br>5= Agree somewhat<br>4= Neither agree or disagree<br>3= Disagree somewhat<br>2= No strongly disagree<br>1= Does not know	5-choice Likert scale: 2 if strongly agree, 0 if<br>strongly disagree with gradations of 0.5<br><br>2 if strongly agree<br>1.5 Agree somewhat<br>1 Neither agree or disagree<br>0.5= Disagree somewhat<br>0= No strongly disagree<br>0= Does not know	2
KAP11	How much danger do you feel that you have of getting sick from dengue fever?	6= All of the time<br>5= Most of the time<br>4= Some of the time<br>3= A little of the time<br>2= None of the time<br>1= Does not know	5-choice Likert scale: 2 if all of the time, 0 if none<br>of the time with gradations of 0.5	2
KAP13	Do you think that your community can prevent dengue on its own?	6= Strongly agree<br>5= Agree somewhat<br>4= Neither agree or disagree<br>3= Disagree somewhat<br>2= No strongly disagree<br>1= Does not know	5-choice Likert scale: 2 if strongly agree, 0 if<br>strongly disagree with gradations of 0.5<br><br>2 if strongly agree<br>1.5 Agree somewhat<br>1 Neither agree or disagree<br>0.5= Disagree somewhat<br>0= No strongly disagree<br>0= Does not know	2

Supplement 1
Questionnaire, KAP score. Household index and Social Capital index

Cross-sectional study on the association between seroprevalence among children and their parents' knowledge, attitudes, and practices in a dengue-endemic city in Bangladesh

Practices (score= 10)				
**#**	**Question**	**Answers**	**Scoring method**	**Score**
KAP8	If you or someone in your family suspects dengue, what do you do?<br><br>*Alternate phrasing/prompting: Do you take anything or get help? If so, what and where?	Do nothing<br>Take headache/fever medicine<br>Increase liquid intake (keep hydrated)<br>Go to the hospital<br>Go to the clinic or see a doctor<br>Go to the pharmacy<br>Other: specify<br>0= Does not know	Sum of the following, divided by 3:<br>2 if it is Go to the hospital or go to the clinic or see a doctor;<br>+1 if the answer is: Take headache/fever medicine, or Increase liquid intake (keep hydrated), or go to the pharmacy<br>0 if the answer is: does not know or do nothing	3
KAP12	In this house, in the last 30 days, what did you/your household do to reduce the presence of mosquitos?	Did nothing<br>Kept water in closed water storage containers<br>Used larvicide in containers<br>Fumigated inside or outside of home<br>Cleaned up breeding grounds (e.g. clean up debris and trash, clean containers, emptying water from containers)<br>Other: specify<br>Does not know<br>Does not want to answer	2 point max<br>3 if it is: Kept water in closed water storage containers, OR Used larvicide in containers, or fumigated inside or outside of home, or cleaned up breeding grounds<br>0 if the answer is: does not know or do nothing.<br><br>*if the sum is higher or equal to 2, the score is 2, otherwise, the score stays the same.	2
KAP14	Since May 2023, have workers from the municipality taken action to control mosquitoes in your home and/or community?	0 No<br>1 Yes<br>0 Don't know	0= Incorrect<br>1= correct	1

KAP15	If mosquito control workers from the municipality came to your house, would you allow them to enter and look for mosquitoes?	1= Yes<br>0 = No<br>0 = Does not know	0= Incorrect<br>1= correct	1

Supplement 1
Questionnaire, KAP score. Household index and Social Capital index

Cross-sectional study on the association between seroprevalence among children and their parents' knowledge, attitudes, and practices in a dengue-endemic city in Bangladesh

1.3 Household index

Variable	Question	Points	Answer	Observation
de3	What type of dwelling is this?	4	Independent	Independent and multi-storied buildings are built from more resistant materials. Independent is higher income. Semi-pucca is made by a combination of mud and brick. Max points 4
		3	Multi-storied	
		2	Semi-pucca	
		1	Slum	
		1	Floating/Rootless	
		0	Other	
de4	What is the main construction material used for the floor of the respondent's house?	3	Cement	Materials are ranked from the most expensive to the least expensive. This not only includes the material but also the labour. Only one answer option. **Max 3 points**
		2	Earth/sand	
		1	Other	
de5	What is the main construction material used for the outer wall of the respondent's house?	1	Cement	Materials are ranked from the most expensive to the least expensive. This includes not only the material but also the labour. Only one answer option. **Max 1 point**
		0	Other material	
de6	What is the main construction material used for the roof?	1	Cement	Materials are ranked from the most expensive to the least expensive. This includes not only the material but also the labour. Only one answer option. **Max 1 point**
		0	Other material	
de7		1	Yes	Max 1 point

	Does your household have a television?	0	No	
de8	Does your household have a refrigerator?	1	Yes	Max 1 point
		0	No	
de9	Does your household have an almirah/wardrobe?	1	Yes	Max 1 point
		0	No	
de10	Does your household have an electric fan?	1	Yes	Max 1 point
		0	No	
de11	How many rooms are there for living in your household?	0-11		More than 4 Rooms = 2 points.
				3 to 2 Rooms= 1 point.
				1 room or less =0
de12	Does this dwelling have a backyard or any external areas?	1	Yes	**Max 1 point**
		0	No	
de15	What are the toilet facilities at your dwelling?	2.5	Safe drainage by flushing/pouring water	One answer option ranked from the most expensive to the least expensive. **Max points 2.5**
		2	Pit latrine with slab/ventilated improved latrine/composting latrine	
		1.5	Unsafe drainage by flushing/pouring water	
		1	Pit latrine without slab/open pit	
		0.5	Raw/open/hanging latrine (permanent/temporary)	
		0	Open defecation/no latrine available	

de16_a	What is the main source of drinking water for members of your household?	2.5	Tap/Piped (Supply) within the home	One answer option ranked from the most expensive to the least expensive. **Max points 2.5**
		2	Tubewell (deep/shallow) within the home	
		1.5	Motor water	
		1	Tubewell (deep/shallow) within the community	
		0.5	Tap/Piped (Supply) within the community	
		0	Bottled water/water jar	
		0	Other	
de16_b	What are your main sources of water for household chores?	2.5	Tap/Piped (Supply) within the home	One answer option ranked from the most expensive to the least expensive. **Max points 2.5**
		2	Tap/Piped (Supply) within the community	
		1.5	Tubewell (deep/shallow) within the home	
		1	Tubewell (deep/shallow) within the community	
		0.5	Bottled water/water jar	
		0	Motor water	
		0	Other	
de_19	Does this residence have an individual water tank?	1	Yes	1 point if they have an individual water tank

1.4 Social capital index

Variable	Question	Answer	Observation
sc1	In the last 12 months, have you or adult members of your household been an active member (regularly do an activity or talk about things) of any of the following types of groups in your community?	1= Community organization (e.g., residents' group)<br>2= Food distribution group<br>3= Political group<br>4= Professional group or union<br>5= Sports/social group<br>6= Religious group<br>7= Women's group<br>8= Committees for health, water, or electricity<br>9= School groups<br>10= My family or I are not involved in any group<br>96= Other: specify<br>99= Does not know<br>98= Does not want to answer	1 point if: Community organization (e.g., residents' group) or, Food distribution group, or Political group, or Professional group or union, or Sports/social group, Religious group, Women's group, or Committees for health, water, or electricity, or School groups.<br><br>0 points otherwise.

sc2	In the last 12 months, did you or other adult members of your household receive from the group any emotional help, economic help or assistance in helping you know or do things	1= Community organization (e.g., residents' group)<br>2= Food distribution group<br>3= Political group<br>4= Professional group or union<br>5= Sports/social group<br>6= Religious group<br>7= Women's group<br>8= Committees for health, water, or electricity<br>9= School groups<br>10= My family or I are not involved in any group<br>96= Other: specify<br>99= Does not know<br>98= Does not want to answer	1 point if: Community organization (e.g., residents' group) or, Food distribution group, or Political group, or Professional group or union, or Sports/social group, Religious group, Women's group, or Committees for health, water, or electricity, or School groups.<br><br>0 points otherwise.
sc3	Of all these groups to which you or adult members of your household belong, which are the top three most important for the household?	1= Community organization (e.g., residents' group)<br>2= Food distribution group<br>3= Political group<br>4= Professional group or union<br>5= Sports/social group<br>6= Religious group<br>7= Women's group<br>8= Committees for health, water, or electricity<br>9= School groups<br>96= Other: specify<br>99= Does not know<br>98= Does not want to answer	1 point if: Community organization (e.g., residents' group) or, Food distribution group, or Political group, or Professional group or union, or Sports/social group, Religious group, Women's group, or Committees for health, water, or electricity, or School groups.<br><br>0 points otherwise.

sc4	In the last 12 months, have you received any help or support from any of the following, this can be emotional help, economic help or assistance in helping you know or do things?	1=Family members outside the household<br>2=Neighbours<br>3=Friends who are not neighbours<br>4=Community leaders<br>5=Religious leaders<br>6=Politicians<br>7=Government officials/civil service<br>8=Charitable organisations/NGO<br>96=Other: specify<br>10=There are no important groups to this household<br>99=Does not know<br>98=Does not want to answer	1 point if: Family members outside the household, or neighbours, friends who are not neighbours, or community leaders, or religious leaders, or politicians, government officials/civil service, or charitable organisations/NGO.<br><br>0 points otherwise.
sc5	In general, do you agree or disagree with the following statement:Most people in this neighborhood are willing to help if you need it.	1= Strongly agree<br>2= Agree somewhat<br>3=Neither agree or disagree<br>4= Disagree somewhat<br>5= Disagree strongly<br>6= Does not know<br>99= Does not want to answer	2 points if: strongly agree<br>1.5 points if: Agree somewhat<br>1 point if: Neither agree or disagree<br>0.5 points if: Disagree somewhat<br>0 points if: Strongly disagree<br>0 points if:Does not know
sc6	In general, do you agree or disagree with the following statement:<br><br>The majority of people in this community generally get along with each other.	1= Strongly agree<br>2= Agree somewhat<br>3=Neither agree or disagree<br>4= Disagree somewhat<br>5= Disagree strongly<br>6= Does not know<br>99= Does not want to answer	2 points if: strongly agree<br>1.5 points if: Agree somewhat<br>1 point if: Neither agree or disagree<br>0.5 points if: Disagree somewhat<br>0 points if: Strongly disagree<br>0 points if:Does not know

sc8	In general, do you agree or disagree with the following statement:<br><br>You feel that you are really part of the community.	1= Strongly agree<br>2= Agree somewhat<br>3=Neither agree or disagree<br>4= Disagree somewhat<br>5= Disagree strongly<br>6= Does not know<br>99= Does not want to answer	2 points if: strongly agree<br>1.5 points if: Agree somewhat<br>1 point if: Neither agree or disagree<br>0.5 points if: Disagree somewhat<br>0 points if: Strongly disagree<br>0 points if:Does not know
sc10	In the last 12 months, have you joined together with other community members to address a common problem or issue?	1= Yes<br>0= No<br>99= Does not know<br>Does not want to answer	1 If yes, otherwise 0
sc11	In the past 12 months, have you talked with a local authority or governmental organization about problems in this community?	1= Yes<br>0= No<br>99= Does not know<br>Does not want to answer	1 If yes, otherwise 0
sc12	In general, how do you feel about the security in your neighborhood?	1= Very safe<br>2= Moderately safe<br>3= Neither safe or unsafe<br>4= Moderately unsafe<br>5= Very unsafe<br>99= Does not know<br>Does not want to answer	2 points if: very safe<br>1.5 points if: moderately safe<br>1 point if: Neither safe or unsafe<br>0.5 points if: Moderately unsafe<br>0 points if: Very unsafe<br>0 points if: Does not know or does not want ot answer
