# Supplementary material for: Cross-sectional study on the association between seroprevalence among children and their parents’ knowledge, attitudes, and practices in a dengue-endemic city in Bangladesh
Source: PLoS Negl Trop Dis. 2026 Oct 21;20(10):e0014802. doi: 10.1371/journal.pntd.0014802 (PMC13712692; doi:10.1371/journal.pntd.0014802)
Supplement: Supplementary file 2 [file pntd.0014802.s002.pdf]

Supplement 2
Seroprevalence

Cross-sectional study on the association between seroprevalence among children and their parents' knowledge, attitudes, and practices in a dengue-endemic city in Bangladesh

Table S2.1 Crude and adjusted seroprevalence in children

	Overall (n =333)	Crude Prevalence (n =333)	95% CI	Adjusted Prevalence (n =333)	95% CI	p-value
**Recent infection (IgM)**						
Yes	31 (9.3%)	**0.09**	0.06, 0.13	**0.05**	0.02, 0.09	< 0.001
No	302 (73.3%)					
**Previous infection (IgG)**						
Yes	26 (7.8%)	**0.07**	0.05, 0.11	**0.03**	0.01, 0.08	< 0.001
No	307 (92.2%)					
**Seropositive (IgM + IgG)**						
Yes	57 (17.1%)	**0.17**	0.13, 0.22	**0.14**	0.10, 0.19	< 0.001
No	276 (82.9%)					

Supplement 2
Seroprevalence

Cross-sectional study on the association between seroprevalence among children and their parents' knowledge, attitudes, and practices in a dengue-endemic city in Bangladesh

Table S2.2 Description of seroprevalence and other characteristics among the four locations and gender

	Uttara		Mirpur		Mugda		Chawkbazaar	
	Male (N=57)	Female (N=58)	Male (N=59)	Female (N=55)	Male (N=49)	Female (N=41)	Male (N=41)	Female (N=52)
**Age of the child**								
Median [Min, Max]	5.91 [2.00, 13.0]	7.47 [2.31, 13.0]	6.11 [2.22, 12.9]	7.94 [2.05, 12.9]	6.05 [2.11, 12.9]	6.18 [2.09, 12.8]	5.96 [2.01, 12.6]	6.77 [2.02, 12.1]
**School attendance**								
No	32 (56.1%)	22 (37.9%)	23 (39.0%)	22 (40.0%)	20 (40.8%)	16 (39.0%)	22 (53.7%)	25 (48.1%)
Yes	25 (43.9%)	36 (62.1%)	36 (61.0%)	33 (60.0%)	29 (59.2%)	25 (61.0%)	19 (46.3%)	27 (51.9%)
**Chronic disease**								
No	57 (100%)	58 (100%)	59 (100%)	55 (100%)	45 (91.8%)	41 (100%)	37 (90.2%)	51 (98.1%)
Yes	0 (0%)	0 (0%)	0 (0%)	0 (0%)	4 (8.2%)	0 (0%)	4 (9.8%)	1 (1.9%)
**Previous dengue episode since 2023**								
No	55 (96.5%)	56 (96.6%)	59 (100%)	55 (100%)	44 (89.8%)	37 (90.2%)	39 (95.1%)	50 (96.2%)
Yes	2 (3.5%)	2 (3.4%)	0 (0%)	0 (0%)	5 (10.2%)	4 (9.8%)	2 (4.9%)	2 (3.8%)
**Seropositive (n =333)**								
No	28 (49.1%)	31 (53.4%)	41 (69.5%)	38 (69.1%)	33 (67.3%)	29 (70.7%)	33 (80.5%)	43 (82.7%)
Yes	12 (21.1%)	13 (22.4%)	3 (5.1%)	5 (9.1%)	8 (16.3%)	8 (19.5%)	5 (12.2%)	3 (5.8%)

Table S2.3 Characteristics of the children who did not take part in the serology evaluation

	Children who did not take the test (N=79)	Children who took the test (N=333)	p-value
**Gender**			
Female	36 (45.6%)	170 (51.1%)	0.857
Male	43 (54.4%)	163 (48.9%)	
**Age of the child**			
Mean (SD)	6.95 (3.32)	6.87 (2.98)	0.568
Median [Min, Max]	6.93 [2.15, 13.0]	6.68 [2.00, 13.0]	
**School attendance**			
No	38 (48.1%)	144 (43.2%)	0.75
Yes	41 (51.9%)	189 (56.8%)	
**Chronic disease**			
No	77 (97.5%)	326 (97.9%)	0.875
Yes	2 (2.5%)	7 (2.1%)	
**Previous dengue episode since 2023**			
No	75 (94.9%)	320 (96.1%)	0.91
Yes	4 (5.1%)	13 (3.9%)	
