# Supplementary material for: Cross-sectional study on the association between seroprevalence among children and their parents’ knowledge, attitudes, and practices in a dengue-endemic city in Bangladesh
Source: PLoS Negl Trop Dis. 2026 Oct 21;20(10):e0014802. doi: 10.1371/journal.pntd.0014802 (PMC13712692; doi:10.1371/journal.pntd.0014802)
Supplement: Supplementary file 3 [file pntd.0014802.s003.pdf]

Supplement 3

KAP questionnaire answers

Cross-sectional study on the association between seroprevalence among children and their parents' knowledge, attitudes, and practices in a dengue-endemic city in Bangladesh

Question	Uttara (N=85)	Mirpur (N=81)	Mugda (N=65)	Chawkbazaar (N=66)	Overall (N=297)
**KAP Yes. Have you heard of dengue?**					
Yes	85 (100%)	81 (100%)	65 (100%)	66 (100%)	297 (100%)
**KAP 2. Do you know what dengue is?**					
Dengue is a disease					
No	57 (67.1%)	64 (79.0%)	52 (80.0%)	59 (89.4%)	232 (78.1%)
Yes	28 (32.9%)	17 (21.0%)	13 (20.0%)	7 (10.6%)	65 (21.9%)
Spread by mosquitoes					
No	30 (35.3%)	13 (16.0%)	29 (44.6%)	23 (34.8%)	95 (32.0%)
Yes	55 (64.7%)	68 (84.0%)	36 (55.4%)	43 (65.2%)	202 (68.0%)
Aedes is responsible for spreading					
No	85 (100%)	81 (100%)	56 (86.2%)	59 (89.4%)	281 (94.6%)
Yes	0 (0%)	0 (0%)	9 (13.8%)	7 (10.6%)	16 (5.4%)
I don't know much about it					
No	73 (85.9%)	78 (96.3%)	56 (86.2%)	54 (81.8%)	261 (87.9%)
Yes	12 (14.1%)	3 (3.7%)	9 (13.8%)	12 (18.2%)	36 (12.1%)
Other					
No	85 (100%)	81 (100%)	64 (98.5%)	65 (98.5%)	295 (99.3%)
Yes	0 (0%)	0 (0%)	1 (1.5%)	1 (1.5%)	2 (0.7%)
**KAP 3. What do you think are the main signs and symptoms of dengue?**					
Fever					
No	3 (3.5%)	3 (3.7%)	4 (6.2%)	7 (10.6%)	17 (5.7%)
Yes	82 (96.5%)	78 (96.3%)	61 (93.8%)	59 (89.4%)	280 (94.3%)
Headache					
No	47 (55.3%)	41 (50.6%)	32 (49.2%)	48 (72.7%)	168 (56.6%)
Yes	38 (44.7%)	40 (49.4%)	33 (50.8%)	18 (27.3%)	129 (43.4%)
Bone pain					
No	54 (63.5%)	44 (54.3%)	61 (93.8%)	57 (86.4%)	216 (72.7%)

Supplement 3
KAP questionnaire answers
Cross-sectional study on the association between seroprevalence among children and their parents' knowledge, attitudes, and practices in a dengue-endemic city in Bangladesh

Yes	31 (36.5%)	37 (45.7%)	4 (6.2%)	9 (13.6%)	81 (27.3%)
Sore muscles					
No	50 (58.8%)	57 (70.4%)	39 (60.0%)	46 (69.7%)	192 (64.6%)
Yes	35 (41.2%)	24 (29.6%)	26 (40.0%)	20 (30.3%)	105 (35.4%)
Rash (red spots on skin)					
No	80 (94.1%)	70 (86.4%)	54 (83.1%)	52 (78.8%)	256 (86.2%)
Yes	5 (5.9%)	11 (13.6%)	11 (16.9%)	14 (21.2%)	41 (13.8%)
Stomach pain					
No	68 (80.0%)	61 (75.3%)	62 (95.4%)	63 (95.5%)	254 (85.5%)
Yes	17 (20.0%)	20 (24.7%)	3 (4.6%)	3 (4.5%)	43 (14.5%)
Eye pain					
No	65 (76.5%)	64 (79.0%)	63 (96.9%)	65 (98.5%)	257 (86.5%)
Yes	20 (23.5%)	17 (21.0%)	2 (3.1%)	1 (1.5%)	40 (13.5%)
Nausea and/or vomiting					
No	27 (31.8%)	42 (51.9%)	26 (40.0%)	44 (66.7%)	139 (46.8%)
Yes	58 (68.2%)	39 (48.1%)	39 (60.0%)	22 (33.3%)	158 (53.2%)
Sweating					
No	83 (97.6%)	78 (96.3%)	64 (98.5%)	66 (100%)	291 (98.0%)
Yes	2 (2.4%)	3 (3.7%)	1 (1.5%)	0 (0%)	6 (2.0%)
Bleeding gums					
No	84 (98.8%)	79 (97.5%)	63 (96.9%)	66 (100%)	292 (98.3%)
Yes	1 (1.2%)	2 (2.5%)	2 (3.1%)	0 (0%)	5 (1.7%)
Nosebleeds					
No	84 (98.8%)	73 (90.1%)	62 (95.4%)	66 (100%)	285 (96.0%)
Yes	1 (1.2%)	8 (9.9%)	3 (4.6%)	0 (0%)	12 (4.0%)
Diarrhea					
No	59 (69.4%)	66 (81.5%)	57 (87.7%)	56 (84.8%)	238 (80.1%)
Yes	26 (30.6%)	15 (18.5%)	8 (12.3%)	10 (15.2%)	59 (19.9%)
Coughing					

Supplement 3
KAP questionnaire answers
Cross-sectional study on the association between seroprevalence among children and their parents' knowledge, attitudes, and practices in a dengue-endemic city in Bangladesh

No	77 (90.6%)	77 (95.1%)	56 (86.2%)	58 (87.9%)	268 (90.2%)
Yes	8 (9.4%)	4 (4.9%)	9 (13.8%)	8 (12.1%)	29 (9.8%)
Weakness					
No	53 (62.4%)	54 (66.7%)	48 (73.8%)	57 (86.4%)	212 (71.4%)
Yes	32 (37.6%)	27 (33.3%)	17 (26.2%)	9 (13.6%)	85 (28.6%)
Does not know					
No	80 (94.1%)	79 (97.5%)	63 (96.9%)	62 (93.9%)	284 (95.6%)
Yes	5 (5.9%)	2 (2.5%)	2 (3.1%)	4 (6.1%)	13 (4.4%)
Does not want to answer					
No	84 (98.8%)	81 (100%)	65 (100%)	66 (100%)	296 (99.7%)
Yes	1 (1.2%)	0 (0%)	0 (0%)	0 (0%)	1 (0.3%)
Other					
No	84 (98.8%)	78 (96.3%)	62 (95.4%)	65 (98.5%)	289 (97.3%)
Yes	1 (1.2%)	3 (3.7%)	3 (4.6%)	1 (1.5%)	8 (2.7%)
**KAP 4. In your opinion, how is dengue spread?**					
By mosquitoes					
No	31 (36.5%)	32 (39.5%)	16 (24.6%)	23 (34.8%)	102 (34.3%)
Yes	54 (63.5%)	49 (60.5%)	49 (75.4%)	43 (65.2%)	195 (65.7%)
By contact with infected people					
No	84 (98.8%)	77 (95.1%)	60 (92.3%)	57 (86.4%)	278 (93.6%)
Yes	1 (1.2%)	4 (4.9%)	5 (7.7%)	9 (13.6%)	19 (6.4%)
By unhygienic food					
No	85 (100%)	78 (96.3%)	64 (98.5%)	66 (100%)	293 (98.7%)
Yes	0 (0%)	3 (3.7%)	1 (1.5%)	0 (0%)	4 (1.3%)
By unboiled water					
No	85 (100%)	80 (98.8%)	65 (100%)	66 (100%)	296 (99.7%)
Yes	0 (0%)	1 (1.2%)	0 (0%)	0 (0%)	1 (0.3%)
By bad weather					
No	83 (97.6%)	79 (97.5%)	65 (100%)	66 (100%)	293 (98.7%)

Supplement 3
KAP questionnaire answers
Cross-sectional study on the association between seroprevalence among children and their parents' knowledge, attitudes, and practices in a dengue-endemic city in Bangladesh

Yes	2 (2.4%)	2 (2.5%)	0 (0%)	0 (0%)	4 (1.3%)
By visiting rural/farming areas					
No	85 (100%)	79 (97.5%)	65 (100%)	66 (100%)	295 (99.3%)
Yes	0 (0%)	2 (2.5%)	0 (0%)	0 (0%)	2 (0.7%)
By skin contact					
No	79 (92.9%)	80 (98.8%)	64 (98.5%)	65 (98.5%)	288 (97.0%)
Yes	6 (7.1%)	1 (1.2%)	1 (1.5%)	1 (1.5%)	9 (3.0%)
By intimate contact					
No	81 (95.3%)	81 (100%)	65 (100%)	66 (100%)	293 (98.7%)
Yes	4 (4.7%)	0 (0%)	0 (0%)	0 (0%)	4 (1.3%)
By the air					
No	83 (97.6%)	78 (96.3%)	65 (100%)	66 (100%)	292 (98.3%)
Yes	2 (2.4%)	3 (3.7%)	0 (0%)	0 (0%)	5 (1.7%)
By water					
No	58 (68.2%)	39 (48.1%)	59 (90.8%)	63 (95.5%)	219 (73.7%)
Yes	27 (31.8%)	42 (51.9%)	6 (9.2%)	3 (4.5%)	78 (26.3%)
Does not know					
No	75 (88.2%)	74 (91.4%)	55 (84.6%)	52 (78.8%)	256 (86.2%)
Yes	10 (11.8%)	7 (8.6%)	10 (15.4%)	14 (21.2%)	41 (13.8%)
Does not want to answer					
No	85 (100%)	81 (100%)	65 (100%)	66 (100%)	297 (100%)
Other: specify					
No	84 (98.8%)	76 (93.8%)	63 (96.9%)	66 (100%)	289 (97.3%)
Yes	1 (1.2%)	5 (6.2%)	2 (3.1%)	0 (0%)	8 (2.7%)
**KAP 5. Where in your community do you often see mosquitoes?**					
Water containers in households					
No	59 (69.4%)	38 (46.9%)	43 (66.2%)	48 (72.7%)	188 (63.3%)
Yes	26 (30.6%)	43 (53.1%)	22 (33.8%)	18 (27.3%)	109 (36.7%)
Trash in the community					

Supplement 3
KAP questionnaire answers

Cross-sectional study on the association between seroprevalence among children and their parents' knowledge, attitudes, and practices in a dengue-endemic city in Bangladesh

No	20 (23.5%)	15 (18.5%)	22 (33.8%)	30 (45.5%)	87 (29.3%)
Yes	65 (76.5%)	66 (81.5%)	43 (66.2%)	36 (54.5%)	210 (70.7%)
Sewers in the community					
No	48 (56.5%)	33 (40.7%)	40 (61.5%)	40 (60.6%)	161 (54.2%)
Yes	37 (43.5%)	48 (59.3%)	25 (38.5%)	26 (39.4%)	136 (45.8%)
Vacant lots					
No	78 (91.8%)	74 (91.4%)	62 (95.4%)	61 (92.4%)	275 (92.6%)
Yes	7 (8.2%)	7 (8.6%)	3 (4.6%)	5 (7.6%)	22 (7.4%)
Abandoned houses					
No	69 (81.2%)	67 (82.7%)	59 (90.8%)	62 (93.9%)	257 (86.5%)
Yes	16 (18.8%)	14 (17.3%)	6 (9.2%)	4 (6.1%)	40 (13.5%)
Garbage dumps in the street					
No	53 (62.4%)	46 (56.8%)	47 (72.3%)	56 (84.8%)	202 (68.0%)
Yes	32 (37.6%)	35 (43.2%)	18 (27.7%)	10 (15.2%)	95 (32.0%)
Domestic organic garbage dumps					
No	63 (74.1%)	54 (66.7%)	59 (90.8%)	63 (95.5%)	239 (80.5%)
Yes	22 (25.9%)	27 (33.3%)	6 (9.2%)	3 (4.5%)	58 (19.5%)
Tires					
No	81 (95.3%)	75 (92.6%)	51 (78.5%)	46 (69.7%)	253 (85.2%)
Yes	4 (4.7%)	6 (7.4%)	14 (21.5%)	20 (30.3%)	44 (14.8%)
Car repair shops					
No	85 (100%)	79 (97.5%)	63 (96.9%)	63 (95.5%)	290 (97.6%)
Yes	0 (0%)	2 (2.5%)	2 (3.1%)	3 (4.5%)	7 (2.4%)
Construction sites					
No	84 (98.8%)	81 (100%)	59 (90.8%)	60 (90.9%)	284 (95.6%)
Yes	1 (1.2%)	0 (0%)	6 (9.2%)	6 (9.1%)	13 (4.4%)
Scrap yards					
No	84 (98.8%)	79 (97.5%)	59 (90.8%)	64 (97.0%)	286 (96.3%)
Yes	1 (1.2%)	2 (2.5%)	6 (9.2%)	2 (3.0%)	11 (3.7%)

Supplement 3
KAP questionnaire answers

Cross-sectional study on the association between seroprevalence among children and their parents' knowledge, attitudes, and practices in a dengue-endemic city in Bangladesh

Does not know					
No	83 (97.6%)	81 (100%)	60 (92.3%)	58 (87.9%)	282 (94.9%)
Yes	2 (2.4%)	0 (0%)	5 (7.7%)	8 (12.1%)	15 (5.1%)
Does not want to answer					
No	85 (100%)	81 (100%)	65 (100%)	65 (98.5%)	296 (99.7%)
Yes	0 (0%)	0 (0%)	0 (0%)	1 (1.5%)	1 (0.3%)
Other: specify					
No	84 (98.8%)	81 (100%)	64 (98.5%)	60 (90.9%)	289 (97.3%)
Yes	1 (1.2%)	0 (0%)	1 (1.5%)	6 (9.1%)	8 (2.7%)
**KAP 6. In your opinion, are there ways that dengue can be prevented?**					
No	10 (11.8%)	1 (1.2%)	7 (10.8%)	4 (6.1%)	22 (7.4%)
Yes	74 (87.1%)	76 (93.8%)	57 (87.7%)	60 (90.9%)	267 (89.9%)
Do not know	1 (1.2%)	4 (4.9%)	1 (1.5%)	2 (3.0%)	8 (2.7%)
**KAP 7. Could you tell me what people should do to prevent dengue?**					
Keep water in closed water storage containers					
No	50 (58.8%)	40 (49.4%)	55 (84.6%)	64 (97.0%)	209 (70.4%)
Yes	35 (41.2%)	41 (50.6%)	10 (15.4%)	2 (3.0%)	88 (29.6%)
Use larvicide in containers					
No	83 (97.6%)	76 (93.8%)	65 (100%)	66 (100%)	290 (97.6%)
Yes	2 (2.4%)	5 (6.2%)	0 (0%)	0 (0%)	7 (2.4%)
Fumigate inside or outside of home					
No	60 (70.6%)	63 (77.8%)	61 (93.8%)	63 (95.5%)	247 (83.2%)
Yes	25 (29.4%)	18 (22.2%)	4 (6.2%)	3 (4.5%)	50 (16.8%)
Clean up breeding grounds					
No	26 (30.6%)	23 (28.4%)	14 (21.5%)	11 (16.7%)	74 (24.9%)
Yes	59 (69.4%)	58 (71.6%)	51 (78.5%)	55 (83.3%)	223 (75.1%)
Does not know					
No	76 (89.4%)	77 (95.1%)	57 (87.7%)	59 (89.4%)	269 (90.6%)
Yes	9 (10.6%)	4 (4.9%)	8 (12.3%)	7 (10.6%)	28 (9.4%)

Does not want to answer					
No	85 (100%)	81 (100%)	65 (100%)	66 (100%)	297 (100%)
Other: specify					
No	85 (100%)	78 (96.3%)	62 (95.4%)	64 (97.0%)	289 (97.3%)
Yes	0 (0%)	3 (3.7%)	3 (4.6%)	2 (3.0%)	8 (2.7%)
**KAP 8. If you or someone in your family suspects dengue, what do you do?**					
Do nothing					
No	85 (100%)	81 (100%)	65 (100%)	64 (97.0%)	295 (99.3%)
Yes	0 (0%)	0 (0%)	0 (0%)	2 (3.0%)	2 (0.7%)
Take headache/fever medicine					
No	69 (81.2%)	72 (88.9%)	52 (80.0%)	60 (90.9%)	253 (85.2%)
Yes	16 (18.8%)	9 (11.1%)	13 (20.0%)	6 (9.1%)	44 (14.8%)
Increase liquid intake					
No	73 (85.9%)	71 (87.7%)	53 (81.5%)	61 (92.4%)	258 (86.9%)
Yes	12 (14.1%)	10 (12.3%)	12 (18.5%)	5 (7.6%)	39 (13.1%)
Go to the hospital					
No	1 (1.2%)	1 (1.2%)	6 (9.2%)	8 (12.1%)	16 (5.4%)
Yes	84 (98.8%)	80 (98.8%)	59 (90.8%)	58 (87.9%)	281 (94.6%)
Go to the clinic or see a doctor					
No	69 (81.2%)	58 (71.6%)	58 (89.2%)	63 (95.5%)	248 (83.5%)
Yes	16 (18.8%)	23 (28.4%)	7 (10.8%)	3 (4.5%)	49 (16.5%)
Go to the pharmacy					
No	85 (100%)	76 (93.8%)	65 (100%)	66 (100%)	292 (98.3%)
Yes	0 (0%)	5 (6.2%)	0 (0%)	0 (0%)	5 (1.7%)
Other: specify					
No	84 (98.8%)	81 (100%)	65 (100%)	64 (97.0%)	294 (99.0%)
Yes	1 (1.2%)	0 (0%)	0 (0%)	2 (3.0%)	3 (1.0%)
**KAP 9. In your opinion, do you think that dengue is a problem for you and your**					

**household?**					
Yes strongly agree	55 (64.7%)	61 (75.3%)	50 (76.9%)	64 (97.0%)	230 (77.4%)
Yes somewhat agree	23 (27.1%)	16 (19.8%)	7 (10.8%)	1 (1.5%)	47 (15.8%)
Neither yes or no	3 (3.5%)	2 (2.5%)	1 (1.5%)	0 (0%)	6 (2.0%)
No somewhat disagree	1 (1.2%)	1 (1.2%)	2 (3.1%)	0 (0%)	4 (1.3%)
No strongly disagree	0 (0%)	0 (0%)	3 (4.6%)	1 (1.5%)	4 (1.3%)
Does not know	3 (3.5%)	1 (1.2%)	2 (3.1%)	0 (0%)	6 (2.0%)
**KAP 10. What are your and your family’s main sources of information about mosquito control and dengue?**					
Family members or friends					
No	40 (47.1%)	39 (48.1%)	40 (61.5%)	42 (63.6%)	161 (54.2%)
Yes	45 (52.9%)	42 (51.9%)	25 (38.5%)	24 (36.4%)	136 (45.8%)
Doctor, nurse					
No	75 (88.2%)	63 (77.8%)	48 (73.8%)	59 (89.4%)	245 (82.5%)
Yes	10 (11.8%)	18 (22.2%)	17 (26.2%)	7 (10.6%)	52 (17.5%)
School					
No	79 (92.9%)	79 (97.5%)	64 (98.5%)	63 (95.5%)	285 (96.0%)
Yes	6 (7.1%)	2 (2.5%)	1 (1.5%)	3 (4.5%)	12 (4.0%)
Entomological/dengue agent					
No	83 (97.6%)	79 (97.5%)	65 (100%)	66 (100%)	293 (98.7%)
Yes	2 (2.4%)	2 (2.5%)	0 (0%)	0 (0%)	4 (1.3%)
Health agent					
No	78 (91.8%)	70 (86.4%)	62 (95.4%)	66 (100%)	276 (92.9%)
Yes	7 (8.2%)	11 (13.6%)	3 (4.6%)	0 (0%)	21 (7.1%)
Community leader					
No	81 (95.3%)	75 (92.6%)	65 (100%)	66 (100%)	287 (96.6%)
Yes	4 (4.7%)	6 (7.4%)	0 (0%)	0 (0%)	10 (3.4%)
Religious leader					
No	85 (100%)	78 (96.3%)	65 (100%)	66 (100%)	294 (99.0%)
Yes	0 (0%)	3 (3.7%)	0 (0%)	0 (0%)	3 (1.0%)

Supplement 3
KAP questionnaire answers
Cross-sectional study on the association between seroprevalence among children and their parents' knowledge, attitudes, and practices in a dengue-endemic city in Bangladesh

Television					
No	23 (27.1%)	22 (27.2%)	13 (20.0%)	18 (27.3%)	76 (25.6%)
Yes	62 (72.9%)	59 (72.8%)	52 (80.0%)	48 (72.7%)	221 (74.4%)
Newspapers					
No	85 (100%)	77 (95.1%)	63 (96.9%)	62 (93.9%)	287 (96.6%)
Yes	0 (0%)	4 (4.9%)	2 (3.1%)	4 (6.1%)	10 (3.4%)
Radio					
No	85 (100%)	80 (98.8%)	65 (100%)	66 (100%)	296 (99.7%)
Yes	0 (0%)	1 (1.2%)	0 (0%)	0 (0%)	1 (0.3%)
Social media and messaging					
No	81 (95.3%)	66 (81.5%)	53 (81.5%)	54 (81.8%)	254 (85.5%)
Yes	4 (4.7%)	15 (18.5%)	12 (18.5%)	12 (18.2%)	43 (14.5%)
Internet searches/browsing					
No	66 (77.6%)	65 (80.2%)	60 (92.3%)	54 (81.8%)	245 (82.5%)
Yes	19 (22.4%)	16 (19.8%)	5 (7.7%)	12 (18.2%)	52 (17.5%)
Does not know					
No	84 (98.8%)	80 (98.8%)	64 (98.5%)	66 (100%)	294 (99.0%)
Yes	1 (1.2%)	1 (1.2%)	1 (1.5%)	0 (0%)	3 (1.0%)
Does not want to answer					
No	85 (100%)	81 (100%)	65 (100%)	66 (100%)	297 (100%)
Other: specify					
No	78 (91.8%)	75 (92.6%)	58 (89.2%)	63 (95.5%)	274 (92.3%)
Yes	7 (8.2%)	6 (7.4%)	7 (10.8%)	3 (4.5%)	23 (7.7%)
**KAP 11. How much danger do you feel that you have of getting sick from dengue fever?**					
All of the time	24 (28.2%)	27 (33.3%)	22 (33.8%)	27 (40.9%)	100 (33.7%)
Most of the time	38 (44.7%)	34 (42.0%)	7 (10.8%)	6 (9.1%)	85 (28.6%)
Some of the time	10 (11.8%)	8 (9.9%)	8 (12.3%)	7 (10.6%)	33 (11.1%)
A little of the time	8 (9.4%)	6 (7.4%)	4 (6.2%)	7 (10.6%)	25 (8.4%)
None of the time	4 (4.7%)	3 (3.7%)	23 (35.4%)	18 (27.3%)	48 (16.2%)

Supplement 3
KAP questionnaire answers
Cross-sectional study on the association between seroprevalence among children and their parents' knowledge, attitudes, and practices in a dengue-endemic city in Bangladesh

Does not know	1 (1.2%)	3 (3.7%)	1 (1.5%)	1 (1.5%)	6 (2.0%)
**KAP 12. In this house, in the last 30 days, what did you/your household do to reduce the presence of mosquitos?**					
Did nothing					
No	77 (90.6%)	73 (90.1%)	37 (56.9%)	33 (50.0%)	220 (74.1%)
Yes	8 (9.4%)	8 (9.9%)	28 (43.1%)	33 (50.0%)	77 (25.9%)
Kept water in closed water storage containers					
No	62 (72.9%)	56 (69.1%)	60 (92.3%)	65 (98.5%)	243 (81.8%)
Yes	23 (27.1%)	25 (30.9%)	5 (7.7%)	1 (1.5%)	54 (18.2%)
Used larvicide in containers					
No	84 (98.8%)	81 (100%)	65 (100%)	66 (100%)	296 (99.7%)
Yes	1 (1.2%)	0 (0%)	0 (0%)	0 (0%)	1 (0.3%)
Fumigated inside or outside of home					
No	33 (38.8%)	40 (49.4%)	46 (70.8%)	48 (72.7%)	167 (56.2%)
Yes	52 (61.2%)	41 (50.6%)	19 (29.2%)	18 (27.3%)	130 (43.8%)
Cleaned up breeding grounds					
No	42 (49.4%)	40 (49.4%)	59 (90.8%)	63 (95.5%)	204 (68.7%)
Yes	43 (50.6%)	41 (50.6%)	6 (9.2%)	3 (4.5%)	93 (31.3%)
Does not know					
No	85 (100%)	80 (98.8%)	64 (98.5%)	65 (98.5%)	294 (99.0%)
Yes	0 (0%)	1 (1.2%)	1 (1.5%)	1 (1.5%)	3 (1.0%)
Does not want to answer					
No	85 (100%)	81 (100%)	65 (100%)	66 (100%)	297 (100%)
Other: specify					
No	85 (100%)	73 (90.1%)	52 (80.0%)	54 (81.8%)	264 (88.9%)
Yes	0 (0%)	8 (9.9%)	13 (20.0%)	12 (18.2%)	33 (11.1%)
**KAP 13. Do you think that your community can prevent dengue on its own?**					
Yes strongly agree	30 (35.3%)	37 (45.7%)	31 (47.7%)	31 (47.0%)	129 (43.4%)
Yes somewhat agree	52 (61.2%)	35 (43.2%)	17 (26.2%)	20 (30.3%)	124 (41.8%)
Neither yes or no	3 (3.5%)	4 (4.9%)	9 (13.8%)	5 (7.6%)	21 (7.1%)

Supplement 3
KAP questionnaire answers
Cross-sectional study on the association between seroprevalence among children and their parents' knowledge, attitudes, and practices in a dengue-endemic city in Bangladesh

No somewhat disagree	0 (0%)	4 (4.9%)	1 (1.5%)	1 (1.5%)	6 (2.0%)
No strongly disagree	0 (0%)	0 (0%)	4 (6.2%)	7 (10.6%)	11 (3.7%)
Does not know	0 (0%)	1 (1.2%)	3 (4.6%)	2 (3.0%)	6 (2.0%)
**KAP 14. Since May 2023, have workers from the municipality taken action to control mosquitoes in your home and/or community?**					
No	1 (1.2%)	0 (0%)	5 (7.7%)	2 (3.0%)	8 (2.7%)
Yes	83 (97.6%)	81 (100%)	57 (87.7%)	63 (95.5%)	284 (95.6%)
Does not know	1 (1.2%)	0 (0%)	3 (4.6%)	1 (1.5%)	5 (1.7%)
**KAP 15. If mosquito control workers from the municipality came to your house, would you allow them to enter and look for mosquitoes?**					
No	0 (0%)	1 (1.2%)	2 (3.1%)	2 (3.0%)	5 (1.7%)
Yes	84 (98.8%)	79 (97.5%)	61 (93.8%)	62 (93.9%)	286 (96.3%)
Does not know	1 (1.2%)	1 (1.2%)	2 (3.1%)	2 (3.0%)	6 (2.0%)

Supplement 3
KAP questionnaire answers
Cross-sectional study on the association between seroprevalence among children and their parents' knowledge, attitudes, and practices in a dengue-endemic city in Bangladesh

Table 3.2 Regression variables by KAP levels

Variable	Knowledge			Attitudes			Practices		
	**Essential (<7)**	**Intermediate (7 -10.5)**	**Knowledgeable (≥ 10.5)**	**Negative (<3)**	**Neutral (3 - 4.5)**	**Positive (≥ 4.5)**	**Introductory (<3.5)**	**Partial (3.5-5.25)**	**Extensive (≥ 5.25)**
	(N=68)	(N=209)	(N=56)	(N=10)	(N=113)	(N=210)	(N=26)	(N=117)	(N=190)
**Child's previous dengue**									
No	57 (100%)	174 (96.1%)	54 (91.5%)	11 (91.7%)	89 (94.7%)	185 (96.9%)	24 (100%)	85 (95.5%)	176 (95.7%)
Yes	0 (0%)	7 (3.9%)	5 (8.5%)	1 (8.3%)	5 (5.3%)	6 (3.1%)	0 (0%)	4 (4.5%)	8 (4.3%)
**Child age in number**	6.75 [2.06, 13.0]	6.55 [2.09, 12.9]	6.80 [2.12, 12.6]	6.09 [2.67, 11.6]	6.79 [2.06, 12.9]	6.56 [2.09, 13.0]	5.72 [2.15, 11.0]	6.55 [2.06, 12.6]	6.80 [2.12, 13.0]
**Child attends school**									
No	31 (51.7%)	73 (40.8%)	28 (48.3%)	4 (33.3%)	45 (47.9%)	83 (43.5%)	11 (45.8%)	39 (43.8%)	82 (44.6%)
Yes	29 (48.3%)	106 (59.2%)	30 (51.7%)	8 (66.7%)	49 (52.1%)	108 (56.5%)	13 (54.2%)	50 (56.2%)	102 (55.4%)
**Chronic disease of the child**									
No	57 (100%)	176 (97.2%)	57 (96.6%)	11 (91.7%)	91 (96.8%)	188 (98.4%)	22 (91.7%)	87 (97.8%)	181 (98.4%)
Yes	0 (0%)	5 (2.8%)	2 (3.4%)	1 (8.3%)	3 (3.2%)	3 (1.6%)	2 (8.3%)	2 (2.2%)	3 (1.6%)
**Child's gender.**									
Male	21 (35.0%)	95 (53.6%)	35 (59.3%)	5 (41.7%)	45 (47.9%)	101 (52.9%)	12 (50.0%)	43 (48.3%)	96 (52.2%)
Female	36 (63.2%)	86 (46.4%)	24 (40.7%)	7 (58.3%)	49 (52.1%)	90 (47.1%)	12 (50.0%)	46 (51.7%)	88 (47.8%)
**Parent's age median**	31.0 [18.9, 81.3]	32.0 [18.1, 77.2]	33.1 [19.1, 50.2]	32.1 [25.8, 51.1]	32.4 [18.1, 81.3]	33.0 [19.0, 77.2]	29.1 [18.9, 44.1]	32.0 [21.0, 62.2]	33.1 [18.1, 81.3]
**Parent's gender**									

Supplement 3
KAP questionnaire answers

Cross-sectional study on the association between seroprevalence among children and their parents' knowledge, attitudes, and practices in a dengue-endemic city in Bangladesh

Male	7 (11.7%)	30 (16.8%)	12 (20.7%)	1 (8.3%)	21 (22.3%)	27 (14.1%)	0 (0%)	18 (20.2%)	31 (16.8%)
Female	53 (88.3%)	151 (83.4%)	47 (79.7%)	11 (91.7%)	73 (77.7%)	164 (85.9%)	24 (100%)	71 (79.8%)	153 (83.2%)
**Level of education**									
Less than primary school	37 (64.9%)	95 (52.5%)	29 (49.2%)	5 (41.7%)	46 (48.9%)	110 (57.6%)	6 (25.0%)	43 (48.3%)	112 (60.9%)
Secondary or more	20 (35.1%)	86 (47.5%)	30 (50.8%)	7 (58.3%)	48 (51.1%)	81 (42.4%)	18 (75.0%)	46 (51.7%)	72 (39.1%)
**Working status**									
Not working at the moment	3 (5.3%)	15 (8.3%)	10 (16.9%)	1 (8.3%)	5 (5.3%)	22 (11.5%)	1 (4.2%)	7 (7.9%)	20 (10.9%)
Working	54 (94.7%)	166 (91.7%)	49 (83.1%)	11 (91.7%)	89 (94.7%)	169 (88.5%)	23 (95.8%)	82 (92.1%)	164 (89.1%)
**Hospitalization of a family member**									
No	57 (100%)	166 (91.7%)	49 (83.1%)	12 (100%)	87 (92.6%)	173 (90.6%)	23 (95.8%)	81 (91.0%)	168 (91.3%)
Yes	0 (0%)	15 (8.3%)	10 (16.9%)	0 (0%)	7 (7.4%)	18 (9.4%)	1 (4.2%)	8 (9.0%)	16 (8.7%)
**Change of residency in the last 5 years**									
No	34 (59.6%)	125 (69.1%)	48 (81.4%)	5 (41.7%)	54 (57.4%)	148 (77.5%)	15 (62.5%)	49 (55.1%)	143 (77.7%)
Yes	23 (40.4%)	55 (30.4%)	11 (18.6%)	7 (58.3%)	39 (41.5%)	43 (22.5%)	9 (37.5%)	39 (43.8%)	41 (22.3%)
Don't know	0 (0%)	1 (0.6%)	0 (0%)	0 (0%)	1 (1.1%)	0 (0%)	0 (0%)	1 (1.1%)	0 (0%)
**# of people with previous dengue in the household**	0 [0, 1.00]	0 [0, 2.00]	0 [0, 3.00]	0 [0, 0]	0 [0, 3.00]	0 [0, 2.00]	0 [0, 1.00]	0 [0, 1.00]	0 [0, 3.00]
**Household income Index**									
Low	26 (45.6%)	67 (37.0%)	23 (39.0%)	3 (25.0%)	29 (30.9%)	84 (44.0%)	5 (20.8%)	19 (21.3%)	92 (50.0%)

Supplement 3
KAP questionnaire answers

Cross-sectional study on the association between seroprevalence among children and their parents' knowledge, attitudes, and practices in a dengue-endemic city in Bangladesh

Medium	15 (26.3%)	53 (29.3%)	17 (28.8%)	7 (58.3%)	25 (26.6%)	53 (27.7%)	6 (25.0%)	32 (36.0%)	47 (25.5%)
High	16 (28.1%)	61 (33.7%)	19 (32.2%)	2 (16.7%)	40 (42.6%)	54 (28.3%)	13 (54.2%)	38 (42.7%)	45 (24.5%)
**Water storage**									
No storage or closed	52 (91.2%)	168 (92.8%)	41 (69.5%)	11 (91.7%)	85 (90.4%)	165 (86.4%)	24 (100%)	81 (91.0%)	156 (84.8%)
Presence of an open water storage	5 (8.8%)	13 (7.2%)	18 (30.5%)	1 (8.3%)	9 (9.6%)	26 (13.6%)	0 (0%)	8 (9.0%)	28 (15.2%)
**Social Capital**									
Low/Moderate	39 (68.4%)	80 (44.2%)	25 (42.4%)	10 (83.3%)	50 (53.2%)	84 (44.0%)	11 (45.8%)	42 (47.2%)	91 (49.5%)
High	18 (31.6%)	101 (55.8%)	34 (57.6%)	2 (16.7%)	44 (46.8%)	107 (56.0%)	13 (54.2%)	47 (52.8%)	93 (50.5%)
