# Supplementary material for: Cross-sectional study on the association between seroprevalence among children and their parents’ knowledge, attitudes, and practices in a dengue-endemic city in Bangladesh
Source: PLoS Negl Trop Dis. 2026 Oct 21;20(10):e0014802. doi: 10.1371/journal.pntd.0014802 (PMC13712692; doi:10.1371/journal.pntd.0014802)
Supplement: Supplementary file 4 [file pntd.0014802.s004.pdf]

Supplement 4
Correlation and regression models

Cross-sectional study on the association between seroprevalence among children and their parents' knowledge, attitudes, and practices in a dengue-endemic city in Bangladesh

Table S4.1. Chi-square test of serology and KAP domains

	Chi2	df	p-value
Knowledge vs Seropositive	0.01	2	0.99
Attitudes vs Seropositive	1.63	2	0.44
Practices vs Seropositive	1.85	2	0.39
Total KAP vs Seropositive	0.33	2	0.84

Table S4.2 Univariate regression models of serology to KAP

Model 1 KAP score

Variable	RR	95% CI	p-value
**(Intercept)**	0.26	0.06-1.14	0.08
**KAP score (continous)**	0.98	0.90-1.06	0.56

Model 2 KAP levels

Variable	RR	95% CI	p-value
**(Intercept)**	0.21	0.09-0.50	0.00
**KAP (moderate)** (Ref. Low)	0.82	0.33-2.06	0.68
**KAP (high)** (Ref. Low)	0.74	0.27-2.03	0.56

Model 3. KAP by domain scores

Variable	RR	95% CI	p-value
**(Intercept)**	0.30	0.07-1.30	0.11
**Knowledge Score**	0.99	0.88-1.10	0.81
**Attitude Score**	0.80	0.65-0.98	0.03
**Practice Score**	1.11	0.90-1.37	0.31

Model 4. KAP by domain levels

Variable	RR	95% CI	p-value
**(Intercept)**	0.55	0.14-2.16	0.39
**Knowledge (Intermediate)** (Ref. Basic)	1.06	0.58-1.91	0.86
**Knowledge (Knowledgeable)** (Ref. Basic)	1.04	0.47-2.29	0.92
**Attitude (Neutral)** (Ref. Negative)	0.45	0.15-1.37	0.16
**Attitude (Positive)** (Ref. Negative)	0.34	0.12-1.02	0.05
**Practice (Partial)** (Ref. Introductory)	0.53	0.23-1.24	0.14
**Practice (Extensive) (Ref. Introductory)**	0.87	0.41-1.84	0.72

Model 5. Knowledge score

Variable	RR	95% CI	p-value

**(Intercept)**	0.20	0.07-0.52	0.00
**Knowledge Score**	0.98	0.88-1.10	0.78

Model 7. Attitudes score

Variable	RR	95% CI	p-value
**(Intercept)**	0.42	0.17-1.08	0.07
**Attitudes score**	0.82	0.67-1.01	0.06

Model 8. Practices score

Variable	RR	95% CI	p-value
**(Intercept)**	0.13	0.04-0.40	0.00
**Practices score**	1.06	0.86-1.30	0.61

Model 9. Knowledge levels

Variable	RR	95% CI	p-value
**(Intercept)**	0.17	0.10-0.30	0.00
**Knowledge (Intermediate)** (Ref. Basic)	0.99	0.53-1.83	0.97
**Knowledge (Knowledgeable)** (Ref. Basic)	1.02	0.47-2.23	0.96

Model 10. Attitudes score

Variable	RR	95% CI	p-value
**(Intercept)**	0.3	0.12-0.77	0.01
**Attitude (Neutral)** (Ref. Negative)	0.62	0.22-1.73	0.36
**Attitude (Positive)** (Ref. Negative)	0.52	0.19-1.42	0.20

Model 11. Practices score

Variable	RR	95% CI	p-value
**(Intercept)**	0.23	0.11-0.47	0.00
**Practice (Partial)** (Ref. Introductory)	0.59	0.25-1.37	0.22
**Practice (Extensive) (Ref. Introductory)**	0.80	0.37-1.71	0.56
