# Supplementary material for: Cross-sectional study on the association between seroprevalence among children and their parents’ knowledge, attitudes, and practices in a dengue-endemic city in Bangladesh
Source: PLoS Negl Trop Dis. 2026 Oct 21;20(10):e0014802. doi: 10.1371/journal.pntd.0014802 (PMC13712692; doi:10.1371/journal.pntd.0014802)
Supplement: Supplementary file 5 [file pntd.0014802.s005.pdf]

Supplement 5

Cross-sectional study on the association between seroprevalence among children and their parents' knowledge, attitudes, and practices in a dengue-endemic city in Bangladesh.

5.1 STROBE Checklist in reports of *cross-sectional studies* *

	Item No	Recommendation	Page
**Title and abstract**	1	(*a*) Indicate the study's design with a commonly used term in the title or the abstract	1
		(*b*) Provide in the abstract an informative and balanced summary of what was done and what was found	2
**Introduction**			
Background/rationale	2	Explain the scientific background and rationale for the investigation being reported	5-7
Objectives	3	State specific objectives, including any prespecified hypotheses	7
**Methods**			
Study design	4	Present key elements of study design early in the paper	10
Setting	5	Describe the setting, locations, and relevant dates, including periods of recruitment, exposure, follow-up, and data collection	8
Participants	6	(*a*) Give the eligibility criteria, and the sources and methods of selection of participants	8, 10
Variables	7	Clearly define all outcomes, exposures, predictors, potential confounders, and effect modifiers. Give diagnostic criteria, if applicable	12-14
Data sources/ measurement	8	For each variable of interest, give sources of data and details of methods of assessment (measurement). Describe comparability of assessment methods if there is more than one group	*12-14*
Bias	9	Describe any efforts to address potential sources of bias	32
Study size	10	Explain how the study size was arrived at	9-10
Quantitative variables	11	Explain how quantitative variables were handled in the analyses. If applicable, describe which groupings were chosen and why	12-14
Statistical methods	12	(*a*) Describe all statistical methods, including those used to control for confounding	15, 16
		(*b*) Describe any methods used to examine subgroups and interactions	15, 16
		(*c*) Explain how missing data were addressed	15, 16
		(*d*) If applicable, describe analytical methods taking account of sampling strategy	15, 16
		(*e*) Describe any sensitivity analyses	15, 16
**Results**			
Participants	13	(a) Report numbers of individuals at each stage of study—eg numbers potentially eligible, examined for	16, 17

		eligibility, confirmed eligible, included in the study, completing follow-up, and analysed	
		(b) Give reasons for non-participation at each stage	16, 17
		(c) Consider use of a flow diagram	NA
Descriptive data	14	(a) Give characteristics of study participants (eg demographic, clinical, social) and information on exposures and potential confounders	16, 17 and Table 1
		(b) Indicate number of participants with missing data for each variable of interest	16, 17
Outcome data	15	Report numbers of outcome events or summary measures	16-17
Main results	16	(*a*) Give unadjusted estimates and, if applicable, confounder-adjusted estimates and their precision (eg, 95% confidence interval). Make clear which confounders were adjusted for and why they were included	Table 4, Supp # 2 and 4
		(*b*) Report category boundaries when continuous variables were categorized	Table 1, Table 4, page 9
		(*c*) If relevant, consider translating estimates of relative risk into absolute risk for a meaningful time period	
Other analyses	17	Report other analyses done—eg analyses of subgroups and interactions, and sensitivity analyses	Supp Material 2 and 3
**Discussion**			
Key results	18	Summarise key results with reference to study objectives	31
Limitations	19	Discuss limitations of the study, taking into account sources of potential bias or imprecision. Discuss both direction and magnitude of any potential bias	32
Interpretation	20	Give a cautious overall interpretation of results considering objectives, limitations, multiplicity of analyses, results from similar studies, and other relevant evidence	27-31
Generalisability	21	Discuss the generalisability (external validity) of the study results	32
**Other information**			
Funding	22	Give the source of funding and the role of the funders for the present study and, if applicable, for the original study on which the present article is based	34

*Completed STROBE checklist for cross-sectional studies. The checklist is from the STROBE statement (von Elm E, Altman DG, Egger M, Pocock SJ, Gotzsche PC, Vandenbroucke JP. The Strengthening the Reporting of Observational Studies in Epidemiology (STROBE) Statement: guidelines for reporting observational studies. PLoS Med 4(10): e296. https://doi.org/10.1371/journal.pmed.0040296), available at https://www.strobe-statement.org/checklists/ , and is distributed under the Creative Commons Attribution (CC BY 4.0) license (https://creativecommons.org/licenses/by/4.0/)

5.2 ChecKAP List

	**ChecKAP: KAP-Reporting Checklist—Checklist of items that should be included in reports of *KAP studies*****		
**Items**	**Item No#**	**ITEM RECOMMENDATION**	Page
	**Title**<br>**General Consideration:** the title should include the fact of reporting a CAP study, the subject of the KAP and possibly the population studied.		**1**
**Title**	**T1**	Describe the title identifying Knowledge, Attitude, and Practice or acronym (KAP) study	1
	**Abstract**<br>**General Consideration:** An informative abstract is a structured abstract with the headings of Objective, Method, Result and Conclusion.<br>Typically, a well-structured abstract has a larger section for Results and Methods and a shorter section for Objective and Conclusion in terms of text length. Avoid including statements in the abstract that require references		
**Abstract structure**	**A1**	Objective: Clearly state the purpose of the study in the objective, without including any background information or need of reasoning.	2
	**A2**	Methods: Briefly describe the main methods used, including the type of study, how the sample was recruited, the tool you used to measure KAP	2
	**A3**	Results: Present the most significant finding related to the objective, along with any other important results. Provide quantitative details, such as p-values or confidence intervals if applicable to your research.	2
	**A4**	Conclusion: Summarize your conclusion without giving recommendations.	3

	. **Keywords**<br>**General Consideration:** The keyword should identify your study as a KAP study. The KAP's subject, the population studied.		
**Key words**	**K1**	Make sure your keywords are listed in MeSH catalog	3
**INTRODUCTION**<br>**General Consideration:** Provide a comprehensive background, rationale, and objective(s) of the KAP study. In the Introduction, you are supposed to show the magnitude of the subject that KAP, the importance of a KAP study in relation to the subject of KAP, the existing knowledge and the gaps of knowledge, and how your objective addresses the gap. The background and introduction section serves as a foundation for the rest of your manuscript, setting the stage for the research you will present. It should engage readers, provide context, and make a clear case for the importance of your KAP study. In the background and introduction section of a manuscript about a Knowledge, Attitudes, and Practices (KAP) study, you should include the following items. Introduction normally does not get a subheading and items can be accommodated in one or more paragraphs.			
**Introduction**	**I1**	**Research Context and Rationale:** Begin by introducing the broader context of your study.<br>The magnitude of the problem (state incidence, prevalence, burden, or other descriptive measures) shows the magnitude of the problem, explains why the specific topic of the KAP study is important and relevant, providing a clear rationale for conducting the research.	**5-7**
	**I2**	**Statement of the Problem:** Clearly state the research problem or question that your KAP study aims to address. This should be a concise statement that outlines the specific area of knowledge, attitudes, and practices that you will be investigating.	**7**
	**I3**	**Significance of the Study:** Explain the significance of your KAP study. Emphasize why understanding the KAP related to the topic is important and how it could contribute to addressing the particular subject that the KAP is about.	**6-7**
	**I4**	**Literature Review:** Provide a brief review of relevant literature. Summarize key studies, findings, and theories related to the topic of your KAP study. This helps to contextualize your research within the existing body of knowledge and demonstrate how your study builds upon or adds to the current understanding.	**5-7**

	I5	**Identify the gap:** Contextualize your research within the existing body of knowledge (the previous item) and demonstrate how your study builds upon or adds to the current understanding. Light on the gaps in knowledge that your study is going to address. Try to be as bold as possible in presenting the gap and how your study is going to address the gap. (This part of the introduction is the part where you are going to convivence reviewer or evaluator that your study merit novelty).	**7**
	I6	**The Objective of the study:** The last paragraph of the introduction should clearly outline the specific objectives of your KAP study. These objectives should outline what you intend to achieve or uncover through your research.	**7**
**METHODS**<br>**General Consideration:** In the method section of a manuscript for a Knowledge, Attitudes, and Practices (KAP) study should be as detailed as possible. The Method should have subheadings.			
**Methods**[1]	**Study design**	Clearly describe the design of the KAP study (e.g., cross-sectional, longitudinal) and its appropriateness for the topic.	9
	**Sampling Technique**	Describe the sampling technique used and its rationale.	10
	**Sample size**	Report the sample size and provide justification for its adequacy.<br>Eligibility criteria for participants, including criteria at different levels in recruitment/sampling plan	9-10
	**Data Collection**	Describe the questionnaire or survey used to assess Knowledge, Attitudes, and Practices, including its development process.	9-10
		Is the KAP measurement tool considered a standard or researcher-developed?<br>In either case, referring to the validity and reliability of the tool is essential.	11
		KAP score:<br>Describe KAP score in detail (eg: Knowledge refers to responders' level of knowledge about [topic/issue/problem].2 ; Attitude refers to responders' feelings towards the subject. 3; Practice refers to the ways in which responder demonstrate their knowledge and attitude through their actions4.	13-14

	**Instrument**	Total KAP score:<br>Description of total KAP score. It is used to rank the level of knowledge, attitude, and practice, and subsequent qualitative analysis was conducted to rank high, medium, and low scores. (e.g., How many scoring points were gained if a person answered all questions correctly?)	13-14
		Detail the data collection procedure, location, duration, and ethical considerations taken. Clearly describe the settings and locations where the data were collected	9,17
	**Main Constructs /Variables**	Clearly define the variables of interest and describe the measurement methods used (e.g., Likert scales, open-ended questions).<br>Identify knowledge (Awareness) gaps, cultural beliefs (Attitude), or behavioral pattern (Practice) as main variables must be assessed	11
	**Data analysis**	Explain the statistical or qualitative analysis methods employed to analyze the KAP data.	NA
	**Ethical consideration**	Describe the ethical considerations undertaken, including obtaining informed consent, ensuring participant confidentiality, and any necessary ethical approvals obtained.	9,17
**RESULTS**<br>***General Consideration****: In the Results section of a manuscript for a Knowledge, Attitudes, and Practices (KAP) study, the following items of content should be included.* (**Maybe all following items has not been mentioned in your manuscript)** but the content should somehow correspond what you explain in the method section.			
**Participant Flow**	**R1**	Describe the flow of participants throughout the study, starting from recruitment and continuing through retention and any exclusions. Clearly detail the number of participants at each stage and any reasons for exclusion.	17, 18
**Participant Characteristics**	**R2**	Provide detailed information about the study participants, including demographic, clinical, and social characteristics. This may encompass age, gender, educational level, socioeconomic status, and any relevant medical history.	17, 18, Table 1

**Reporting of KAP' Findings**	**R3**	**Knowledge Findings:** Present the results related to participants' knowledge in a structured manner. Highlight key findings, trends, and variations in knowledge levels among different participant groups, if applicable. . Support your description with tables or figures. (avoid very simple figures)..	**22-24, Supp 3, table 2**
	**R4**	**Attitudes Findings**: Similarly, present the results related to participants' attitudes in a clear and structured manner. Identify prevalent attitudes, variations, and any factors influencing attitudes within the participant population. Support your description with tables or figures. (avoid very simple figures).	**22-24, Supp 3, table 2**
	**R5**	**Practices Findings:** Present the findings regarding participants' practices in a well-organized way. Describe common practices, deviations from recommended practices, and any factors affecting behavior. . Support your description with tables or figures. (avoid very simple figures).	**22-24, Supp 3, table 2**
**Further analysis**	**R6**	**Correlation or other modeling:** If relevant, analyze and present any correlations or associations between knowledge, attitudes, and practices. Highlight significant relationships or patterns that emerged from the data. Report the results quantitatively, using appropriate statistical measures such as mean, median, percentage, or frequency. Include confidence intervals or p-values when applicable to provide a sense of the significance of the findings	**24**
	**R7**	**Subgroup Analyses:** If the study involved different participant subgroups (e.g., age groups, gender), present separate findings for each subgroup and discuss any differences or similarities observed.	**Supp 3, and table 2**
**DISCUSSION**<br>**General Consideration:** In the discussion section of a manuscript for a Knowledge, Attitudes, and Practices (KAP) study, the following items should be included.			
	**D1**	Qualitatively report the main finding of your study in the first paragraph of the discussion section (build a platform for flow of discussion).	27
	**D2**	Explaining the strengths and weaknesses of the designed tool and comparing it with	32

**KAP results interpretation Scientific justification**		other similar tools	
	**D3**	Sampling Bias: Explain any potential biases in the selection of participants that might affect the generalizability of your findings.	31, 32
	**D4**	Sample Size: Discuss whether the sample size was sufficient to draw meaningful conclusions and address the study's objectives and representative of the target population, underscore how this enhances the reliability of your results.	32
	**D5**	Self-Report Bias: Acknowledge the possibility that participants might not accurately report their knowledge, attitudes, or behaviors, leading to potential inaccuracies.	32
	**D6**	Social Desirability Bias: Mention the chance that participants may provide responses they perceive as socially desirable, rather than their true beliefs or behaviors.	32
	**D7**	Highlight the limitation of a cross-sectional study design pertaining to your KAP study.	32, 33
	**D8**	Recall Bias: Address the potential for participants to recall information inaccurately, especially when it comes to past behaviors or experiences.	31. 32
	**D9**	Cultural Context: state how cultural norms and values might have influenced responses and implication of this influence in your result and conclusion.	30, 32
Strength	**D10**	Holistic Understanding: Highlight how the KAP study design allows for a comprehensive assessment of knowledge, attitudes, and practices, providing a well-rounded perspective.	30
	**D11**	Real-world Application: Emphasize the practical implications of the study's findings, demonstrating how they could contribute to targeted interventions or policies.	31
	**D12**	Structured Methodology: Explain how the study's structured approach to collecting and analyzing data contributes to the rigor and reliability of the findings.	31
	**D13**	Quantitative and Qualitative Insights: If you used both quantitative and qualitative methods, highlight how this combination offers a more sensed understanding of the subject.	NA
	**D14**	Validation Measures If you used validated tools or instruments, mention how this adds validity to the study's outcomes.	11, 31
	**D15**	Report Limitations and Strength of the study at the end of the discussion. To specify the limitation as a headline is not necessary	31-33
	**Conclusion**		

**Conclusion**	**D16**	Conclude the study's result and its implications for practice and for further studies relevant to improve the short coming of your study or direct toward a gap that may exist.	33
**Acknowledgment and ethical consideration**			
**General Consideration:** Please state how the study addressed ethical conduct of the research, any disclosure that maybe need, if conflict of interest, if the study was approved by any scientific body, part of a student thesis, funded by a body, and etc..			**17, 33, 34**

** Zarei, F., Dehghani, A., Ratansiri, A., Ghaffari, M., Raina, S., Halimi, A., Rakhshanderou, S., Isamel, S., Amiri, P., Aminafshar, A., & Mosavi Jarrahi, A. (2024). ChecKAP: A Checklist for Reporting a Knowledge, Attitude, and Practice (KAP) Study. *Asian Pacific Journal of Cancer Prevention*, *25*(7), 2573–2577. https://doi.org/10.31557/APJCP.2024.25.7.2573
